# High-throughput screen *in vitro* identifies dasatinib as a candidate for combinatorial treatment with HER2-targeting drugs in breast cancer

**DOI:** 10.1371/journal.pone.0280507

**Published:** 2023-01-27

**Authors:** Lisa Svartdal Normann, Mads Haugland Haugen, Vesa Hongisto, Miriam Ragle Aure, Suvi-Katri Leivonen, Vessela N. Kristensen, Andliena Tahiri, Olav Engebraaten, Kristine Kleivi Sahlberg, Gunhild Mari Mælandsmo

**Affiliations:** 1 Department of Research and Innovation, Vestre Viken Hospital Trust, Drammen, Norway; 2 Department of Tumor Biology, Institute for Cancer Research, Oslo University Hospital, Oslo, Norway; 3 Department of Medical Genetics, Institute of Clinical Medicine, Faculty of Medicine, University of Oslo and Oslo University Hospital, Oslo, Norway; 4 Division of Toxicology, Misvik Biology, Turku, Finland; 5 Applied Tumor Genomics Research Program, Medical Faculty, University of Helsinki, Helsinki, Finland; 6 Department of Oncology, Oslo University Hospital, Oslo, Norway; 7 Institute of Clinical Medicine, Faculty of Medicine, University of Oslo, Oslo, Norway; 8 Institute for Medical Biology, Faculty of Health Sciences, UiT–The Arctic University of Norway, Tromsø, Norway; Universita degli Studi di Parma, ITALY

## Abstract

Human epidermal growth factor receptor 2-positive (HER2+) breast cancer is an aggressive subtype of this disease. Targeted treatment has improved outcome, but there is still a need for new therapeutic strategies as some patients respond poorly to treatment. Our aim was to identify compounds that substantially affect viability in HER2+ breast cancer cells in response to combinatorial treatment. We performed a high-throughput drug screen of 278 compounds in combination with trastuzumab and lapatinib using two HER2+ breast cancer cell lines (KPL4 and SUM190PT). The most promising drugs were validated *in vitro* and *in vivo*, and downstream molecular changes of the treatments were analyzed. The screen revealed multiple drugs that could be used in combination with lapatinib and/or trastuzumab. The Src-inhibitor dasatinib showed the largest combinatorial effect together with lapatinib in the KPL4 cell line compared to treatment with dasatinib alone (*p* < 0.01). *In vivo*, only lapatinib significantly reduced tumor growth (*p* < 0.05), whereas dasatinib alone, or in combination with lapatinib, did not show significant effects. Protein analyses of the treated xenografts showed significant alterations in protein levels compared to untreated controls, suggesting that all drugs reached the tumor and exerted a measurable effect. *In silico* analyses suggested activation of apoptosis and reduced activity of survival pathways by all treatments, but the opposite pattern was observed for the combinatorial treatment compared to lapatinib alone.

## Introduction

HER2+ breast cancer is an aggressive form of cancer affecting about 15–20% of all breast cancer patients [1, 2]. Gene amplification of *ERBB2* or overexpression of the transmembrane tyrosine kinase receptor HER2 results in constitutively active downstream cellular signaling pathways and excessive proliferation, growth, and disease progression [3]. Over the past two decades, multiple HER2-targeting drugs have reached the clinic, improving the outcome for this patient group. Such drugs include monoclonal antibodies targeting the extracellular domain of HER2, e.g., trastuzumab, pertuzumab and margetuximab. Furthermore, several tyrosine kinase inhibitors (TKIs) targeting HER2 are in clinical use including lapatinib, neratinib and tucatinib. Newer targeted drugs combine cytotoxic compounds linked to HER2-directed antibodies, such as trastuzumab-emtansine and trastuzumab-deruxtecan.

Despite the improved survival rates for HER2+ breast cancer patients, drug resistance still occurs, leading to relapse and ultimately death. There is therefore a need to develop new treatment strategies to improve current treatment outcomes. High-throughput drug screens in cancer cell lines have been used to identify compounds or drug combinations with the potential to improve the response rate [4]. In this study, we applied a drug screen to two HER2+ breast cancer cell lines. By using cell lines with a low response to trastuzumab and lapatinib, and introducing an additional drug, we aimed to find drug combinations that affect cell viability more effectively. The 278 drugs in the screen were either approved by the Food and Drug Administration (FDA) or evaluated in clinical trials at different stages. The most promising candidates were further tested *in vitro* and *in vivo*. Subsequently, protein analyses were performed to investigate the molecular changes, and *in silico* analyses were used to derive functional effects induced by the treatment combinations.

## Materials and methods

### Cell lines

Two human breast cancer cell lines were used in this study. KPL4 cells were provided by Professor J. Kurebayashi (Kawasaki Medical School, Japan) [5] and SUM190PT cells were provided from Karmanos Cancer Institute in Michigan, USA. Both KPL4 and SUM190PT cells are HER2+, ER negative (ER-) and PIK3CA-mutated [6]. The KPL4 cells were cultured in Dulbecco’s Modified Eagle’s Medium (4.5 g/L glucose) (DMEM; Sigma-Aldrich, St. Louis, MO, USA) supplemented with 10% fetal bovine serum (FBS), 2 mM L-glutamine and 1% penicillin/streptomycin (Sigma-Aldrich) for the high-throughput screen and validation. For synergy experiments and cell culturing before *in vivo* experiments, the KPL4 cells were cultured in DMEM supplemented with 10% FBS, 1% Glutamax and 2% Hepes (Sigma-Aldrich). The SUM190PT cells were cultured in Ham’s F-12 + Glutamax supplemented with 5 μg/mL insulin, 0.1% hydrocortisone, 2.5 μg/mL fungizone, 25 μg/mL gentamicin, 2.5 μg/mL plasmocin, 5 mM ethanolamine, 10 mM Hepes, 5 μg/mL transferrin, 10 nM T3, 50 nM sodium selenite, and 1 g/L Bovine Serum Albumine (Sigma-Aldrich). The cells were routinely tested for mycoplasma infections and cultured at 37°C, 5% CO_2_ for a maximum of 30 passages prior to use. The cell lines were chosen based on their inferior responsiveness to trastuzumab and lapatinib, as previously described [6].

### High-throughput drug screen

To identify drugs that affect HER2+ breast cancer cells in response to targeted treatment with trastuzumab and/or lapatinib, the effects of 278 drugs were screened in KPL4 and SUM190PT cells in 384-well plates. A list of all drugs and their targets can be found in S1 Table. The plates were pre-printed with the drugs in five different concentrations spanning from 0.1 to 10,000 nM. The cells were seeded in the plates (1,500 cells/well) using Multidrop Combi (Thermo Fisher Scientific, Waltham, MA USA) and incubated for 72 hours. Cell viability was measured using CellTiter-Glo Luminescent Cell Viability Assay (CTG; Promega, Madison, WA, USA) according to the provider’s protocol, and output was measured on an Envision plate reader (PerkinElmer, Norwalk, CT, USA).

The drug screen was performed in four different settings: drug screen panel alone, panel plus 10 μg/mL trastuzumab (Roche Applied Biosciences, Basel, Switzerland); panel plus 0.1 μM lapatinib (LC Laboratories, Woburn, MA, USA); and panel plus 10 μg/mL trastuzumab and 0.1 μM lapatinib in combination. The screen was performed in two replicates, resulting in a total of 80 microwell plates used. An overview of the screen setup can be seen in S1 Fig.

The cell viability measurements at the different concentrations were used to calculate a drug sensitivity score (DSS) for each drug to compare the sensitivity between different drugs in the same cell line or between the same drug in different cell lines. The drug sensitivity score was calculated using the area under curve (AUC), the area below the minimum activity level, and the maximal response area, as described by Yadav et al. [7]. We determined a combinatorial effect by calculating the difference in DSS between combinatorial treatments and each drug alone. Half-maximal response values, EC50-values, were calculated for each drug in the panel alone and in combination with trastuzumab and lapatinib.

### *In vitro* models and synergy testing

KPL4 and SUM190PT cells were treated with carboplatin (The National Cancer Institute Developmental Therapeutics program (NCI DTP)), CUDC-101 (SelleckChem, Houston, TX, USA), danusertib (SelleckChem), dasatinib (LC Laboratories), OSI-906 (linsitinib; ChemieTek, Indianapolis, IN, USA), navitoclax (SelleckChem), GDC-0941 (pictilisib; LC Laboratories), prednisone (ENZO, Farmingdale, NY, USA) and quisinostat (JNJ-26481585; Active Biochem LTD, Kowloon, Hong Kong) in eight different concentrations (0.000256:20 μM) in 384-well plates. The cells were treated with the drugs alone and in combination with trastuzumab (10 μg/mL; Roche Applied Biosciences) and lapatinib (0.1 μM; LC Laboratories). The cells were incubated with the drugs for 72 hours before viability measurements using CTG solution (Promega) and signal scanning on Envision plate reader (PerkinElmer). The experiment was conducted with four replicates of each concentration. The setup was repeated twice. Differences between treatment groups were calculated using Student’s t-test.

KPL4 cells were treated with tucatinib, venetoclax and dasatinib (SelleckChem) in three different concentrations in 96-well plates (Costar 96-well white, clear-bottom polystyrene plates, Corning Inc., Corning, NY, USA). Cell viability was measured using a CTG assay (Promega) and readout on a Victor X plate reader (PerkinElmer) after 72 hours. Each experiment was performed in three biological replicates, and differences between treatment groups were calculated using Student’s t-test.

To determine possible synergy, experiments combining treatments of two and two drugs, each in three different concentrations, were conducted. Viability data for each combination was uploaded to the CalcuSyn software (BioSoft, Cambridge, United Kingdom), which provided combination index values (CI) and a predicted effect on synergy (CI < 1), additivity (CI 0.9–1.1) or antagonism (CI > 0.9) based on Chou and Talalay [8].

### *In vivo* models

Female, athymic nude *foxn1*^*nu*^ mice were bred at the Department of Comparative Medicine, Oslo University Hospital, the Norwegian Radium Hospital. Food and water were supplied *ad libitum*, and the mice were kept in a pathogen-free environment at a constant temperature (22±1°C) and humidity (62±5%); 15 air changes/h and a 12-h light/dark cycle. The animals were 6–8 weeks old, and their weight 20–25 g when included in experiments. Subcutaneous injection of cells and ear-markings were performed under inhalation anesthesia using Sevofluran (Baxter, Deerfield, IL, USA). For the induction chamber, the flow rates of O_2_ and N_2_O were 1 l/min and 3 l/min, respectively, and the concentration of Sevofluran was 5% (v/v). For the mask, the flow rate of O_2_ was 0.5 l/min with 3–3.5% Sevofluran. For environmental enrichment, the mice were given nesting material in the form of paper and houses made of cardboard. All protocols were designed, and procedures conducted, in accordance with the guidelines of the Federation of European Laboratory Animal Science Association. The experiments were approved by the Norwegian Food Safety Authority (FOTS numbers 11731 and 15368).

In the first animal experiment, KPL4 cells were resuspended in PBS (Sigma-Aldrich) and Matrigel (VWR, Wayne, PA, USA) 1:1 and kept on ice until injection. Three million cells were injected into mammary fat pad number two, unilateral tumors. The tumors were grown to approximately 150 mm^3^ before treatment start. The animals were randomized into four groups (*n* = 6 mice per group) and treated with tucatinib (25 mg/kg) or venetoclax (25 mg/kg; SelleckChem) either alone or in combination. The drugs were dissolved in DMSO (Merck, Kenilworth, NJ, USA), PEG300, Tween80 (Sigma-Aldrich) and ddH_2_O, and the control group received only vehicle solution. The treatment was given via oral gavage five days per week for two weeks. The mice were monitored daily for sign of illness and deviation from normal behavior. Body weight was registered daily and the tumor width (W) and length (L) were measured twice weekly using a caliper. The volume (V) was calculated as follows: V = W^2^ × L × 0.5. The mice were euthanized by cervical dislocation at end of experiment, if the tumor reached the maximum allowable size (>15 mm in one dimension, or volume >1500 mm^3^), or if total weight loss exceed 10%.

In the second animal experiment, KPL4 cells were resuspended in PBS and kept on ice. Nude mice were injected with 2.5 million KPL4 cells in mammary fat pad number two, bilateral tumors. The tumors were grown to approximately 90 mm^3^ before treatment. One animal displaying a tumor with a volume < 40 mm^3^ at the start of treatment was excluded. The animals were randomized into four groups (*n* = 6 mice per group) and treated with dasatinib (10 mg/kg; SelleckChem; dissolved in DMSO, PEG300, Tween80, ddH_2_O) and lapatinib (100 mg/kg; dissolved in DMSO, PEG300, H_2_O) alone or in combination. The control group received only vehicle solution. The treatment was administered by daily oral gavage for 37 days. The tumors were measured twice weekly using a caliper. On day 38, 24 hours after the last treatment, the animals were euthanized by cervical dislocation and tumors were harvested (three tumors per group, two from the same animal). The tumor pieces were snap frozen in liquid nitrogen and stored at -80°C.

### Reverse phase protein array and statistical analysis

Snap frozen tumor tissues from the *in vivo* experiments were physically ground with a mortar and dissolved using a lysis buffer. The buffer contained Triton X-100, EGTA, Na_3_VO_4_, Hepes (Sigma-Aldrich), NaCl (Merck), MgCl_2_, Na_4_O_7_P_2_ (Fluka Chemie GmbH, Buchs, Switzerland), Glycerol (GE Healthcare, Chicago, IL, USA), PhosStop Phosphatase Inhibitor Cocktail Tablets, and cOmplete Protease Inhibitor Cocktail Tablets (Roche Applied Biosciences). Protein concentrations were determined using Pierce BCA Protein Assay Kit (Thermo Fisher Scientific) according to the manufacturer’s protocol and measured on a Victor X Plate Reader (PerkinElmer). Protein analyses were carried out at the MD Anderson core facility using the Reverse Phase Protein Array (RPPA) method [9]. Internally validated primary antibodies were used, excluding those produced in mice due to potential cross reactivity of the secondary antibodies used for detection with mouse proteins from the xenograft samples. This resulted in 417 antibodies being available for use. Data was analyzed in R (version 4.0.2; R Foundation for Statistical Computing, Vienna, Austria) using R Studio (version 1.1.423) [10]. Normalized linear data was log_2_-transformed and differences in protein levels between treatment groups were analyzed by comparing the log_2_-fold change (FC) values and calculating *p*-values using Student’s t-tests. Volcano plots were prepared to visualize the protein level regulations, and *p* < 0.05 was considered statistically significant. The R package clustermap (version 1.2.3) [11] was used to prepare a heatmap with Pearson average supervised clustering. The data was median centered with tanh adjustment for visualization to compress the extreme values. *In silico* pathway enrichment analyses were performed using the Ingenuity Pathway Analyses (IPA) software (version 57662101; QIAGEN Inc., https://www.qiagenbioinformatics.com/products/ingenuity-pathway-analysis [12]). Genes encoding the proteins were available for enrichment analysis in the IPA software. FC-values and *p*-values of all protein regulations between treatment groups were subjected to core analyses. For visualization purposes, only regulations with a *p* < 0.05 and |FC| > 0.3 are presented in Fig 8.

## Results

### High-throughput screen identified drugs with combinatorial effects in HER2+ cells

**Fig 1:**
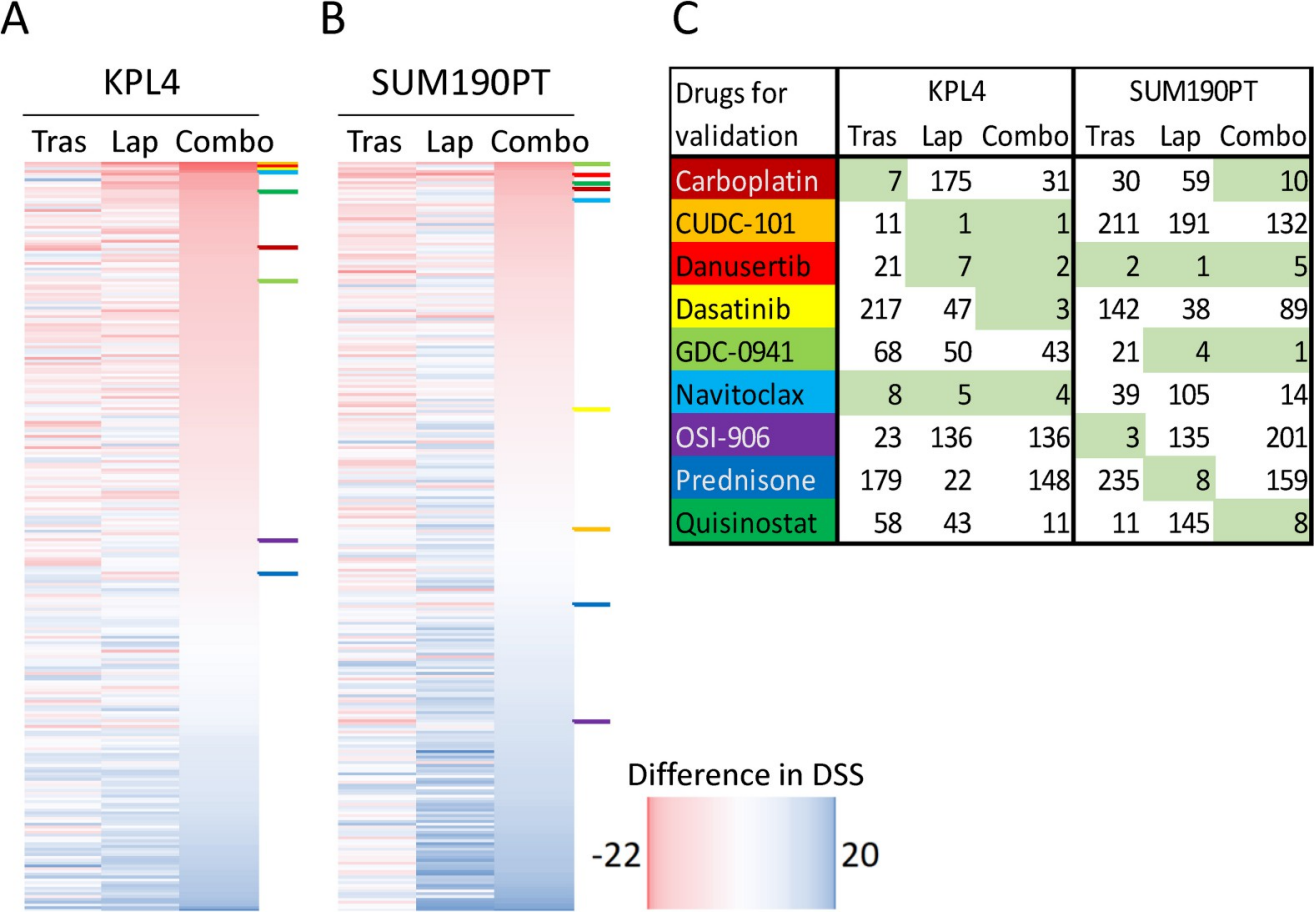
Combinatorial effect of drugs in high-throughput drug screen. Heatmaps displaying the differences in drug sensitivity score (DSS) for 278 drugs screened in combination with trastuzumab (Tras), lapatinib (Lap) and in combination (Combo) compared to drug panel alone in the cell lines KPL4 (A) and SUM190PT (B). The drugs are sorted from the top high (red) to low (blue) combinatorial effect in the respective drug combination column. Combinatorial effect was calculated as the difference in DSS between combination treatment and the drugs alone giving a negative score if high effects were obtained. Panel (C) lists the drugs selected for further validation. Their position in the heatmaps (A+B) is indicated using the corresponding colors. The table shows combinatorial effect rank numbers of the nine selected drugs within each treatment group (among all drugs in the screen). Each selected drug was ranked within top ten in at least one treatment group, as highlighted by the green color.

To identify novel therapeutic combinations with promising effects in two HER2+ breast cancer cell lines, a screen of 278 drugs was performed. We determined the cell viability in cultures treated with each drug in the screen panel alone, and compared it with cultures exposed to the drugs in combination with lapatinib and/or trastuzumab. Overall, the inhibiting effects of the drugs were higher in combination in both KPL4 (Fig 1A) and SUM190PT cells (Fig 1B), indicated by negative difference in DSS, but the KPL4 cells were more prone to the effects of lapatinib, while trastuzumab exerted more effect in SUM190PT cells. Furthermore, a selection of nine drugs from the screen were chosen for further validation based on their ability to reduce cell viability and their functionality. The selected drugs ranked among top ten affecting cell viability in combination with trastuzumab (Tras) or lapatinib (Lap), or in combination with both (Combo) (Fig 1C). The position of these nine drugs in the heatmaps are presented using colors. The viability curves and EC50-values for these nine drugs from the screen are presented in S2 Fig.

### Dasatinib and navitoclax—candidates from the high-throughput screen

**Fig 2:**
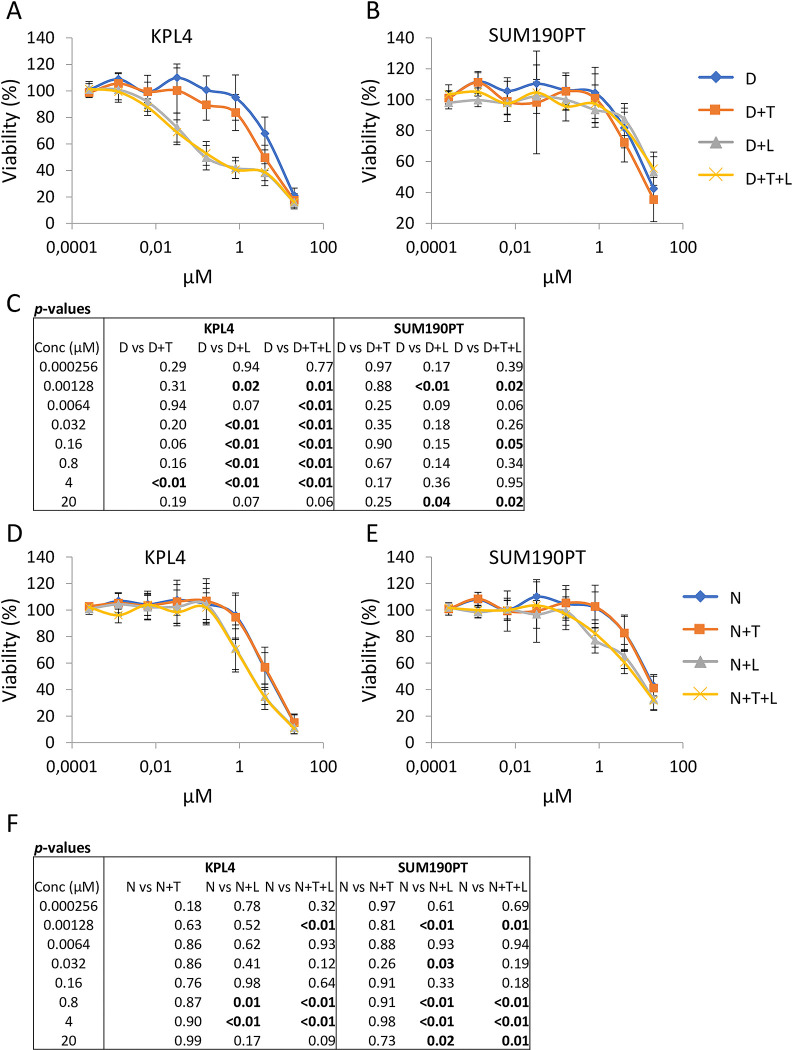
Dose response curves after single drug and combinatorial treatment in HER2+ breast cancer lines. Cell viability measurements after treatment with increasing doses of dasatinib alone and in combination with trastuzumab (10 μg/mL) and lapatinib (0.1 μM) in A) KPL4 cells, and B) SUM190PT cells. Table of *p*-values is presented in panel C). Treatment with increasing doses of navitoclax alone and in combination with trastuzumab (10 μg/mL) and lapatinib (0.1 μM) in D) KPL4 cells, and E) SUM190PT cell. Table of *p*-values is presented in panel F). Student’s t-test *p*-values are calculated between treatment groups at the indicated concentrations. *P-*values < 0.05 are considered significant and are highlighted in bold font. Error bars represent the standard deviation for 4 technical replicates repeated in two biological experiments. D, Dasatinib; L, Lapatinib; T, Trastuzumab; N, Navitoclax.

Nine drug candidates were chosen for further evaluation based on their effect on viability in HER2+ cells in combination with lapatinib and/or trastuzumab compared to each drug from the screen alone. These were carboplatin, CUDC-101, danusertib, dasatinib, OSI-906 (linsitinib), navitoclax, GDC-0941 (pictilisib), prednisone and quisinostat (JNJ-26481585). Their mechanisms of action are listed in S2 Table. The nine candidates were validated *in vitro* in both KPL4 and SUM190PT cells (Fig 2 and S3 Fig). In the validation studies, the TKI dasatinib and the Bcl-x_L_/Bcl-2-targeting drug navitoclax were the most promising candidates and were selected for further investigation. Compared to treatment with the candidate drugs alone, dasatinib showed the largest combinatorial effect with lapatinib, but only in the KPL4 cells (Fig 2A–2C). However, navitoclax in combination with lapatinib showed growth inhibition in both the KPL4 and SUM190PT cell lines (Fig 2D–2F). Trastuzumab did neither improve the effect of the candidate drugs alone, nor in combination with lapatinib.

For five out of eight dasatinib concentrations, the effect of adding lapatinib to dasatinib resulted in significant growth inhibition in the KLP4 cell line (*p* < 0.01, Fig 2A and 2C). In SUM190PT cells, dasatinib treatment and combinatorial treatment with lapatinib and trastuzumab showed modest effects, but the combinations caused significant differences compared to dasatinib alone at some concentrations (*p* < 0.05; Fig 2B and 2C). For navitoclax, cell viability at certain doses (0.00128, 0.8 and 4 μM) was significantly reduced by the addition of lapatinib or lapatinib and trastuzumab in combination in KPL4 cells (*p* < 0.01; Fig 2D and 2F). In SUM190PT cells, the cell viability was significantly reduced by either combining navitoclax and lapatinib or navitoclax together with trastuzumab and lapatinib at concentrations 0.00128, 0.032, 0.8, 4 and 20 μM compared to navitoclax alone (*p* < 0.05; Fig 2E and 2F).

### *In vitro* synergy testing of tucatinib, venetoclax and dasatinib

**Fig 3:**
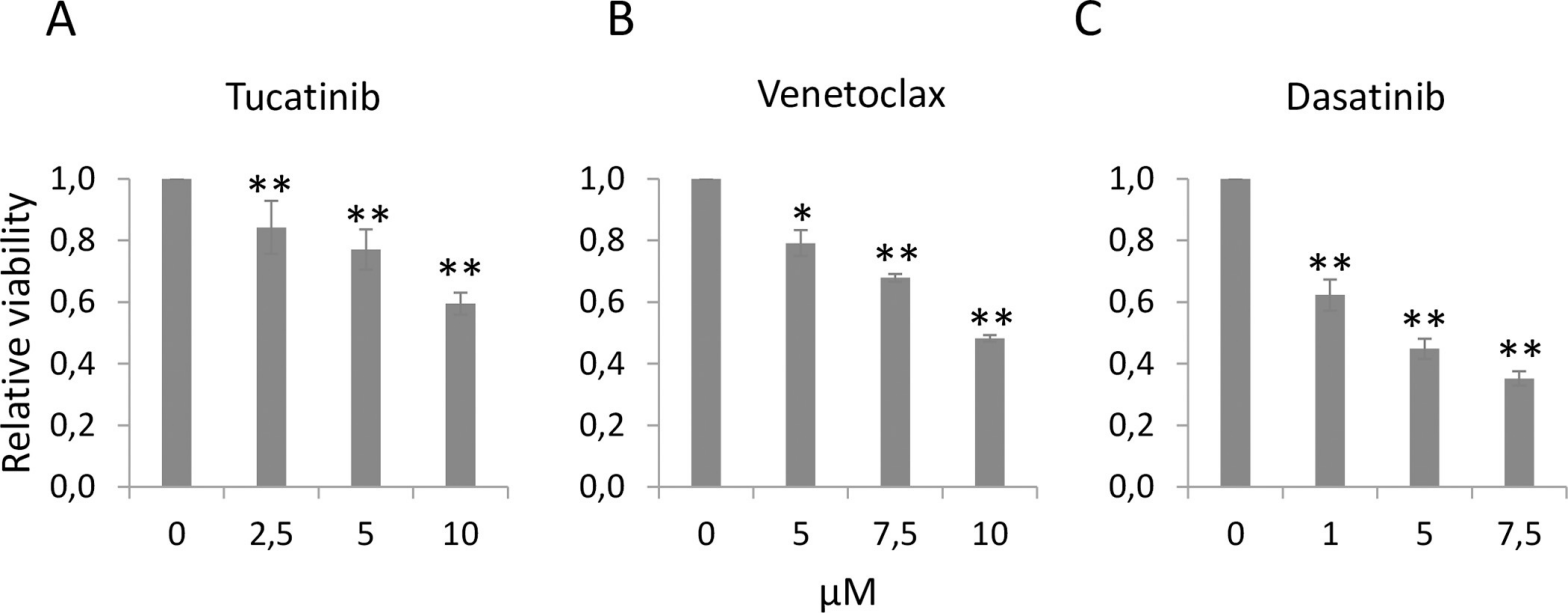
Sensitivity to tucatinib, venetoclax and dasatinib in KPL4 cells. Cells were treated with increasing doses of A) tucatinib, B) venetoclax and C) dasatinib for 72 hours. Cell viability is showed as relative to control. *p < 0.05, **p < 0.01, Student’s t-test. Error bars represent the standard deviation of ≥ 3 biological experiments.

**Fig 4:**
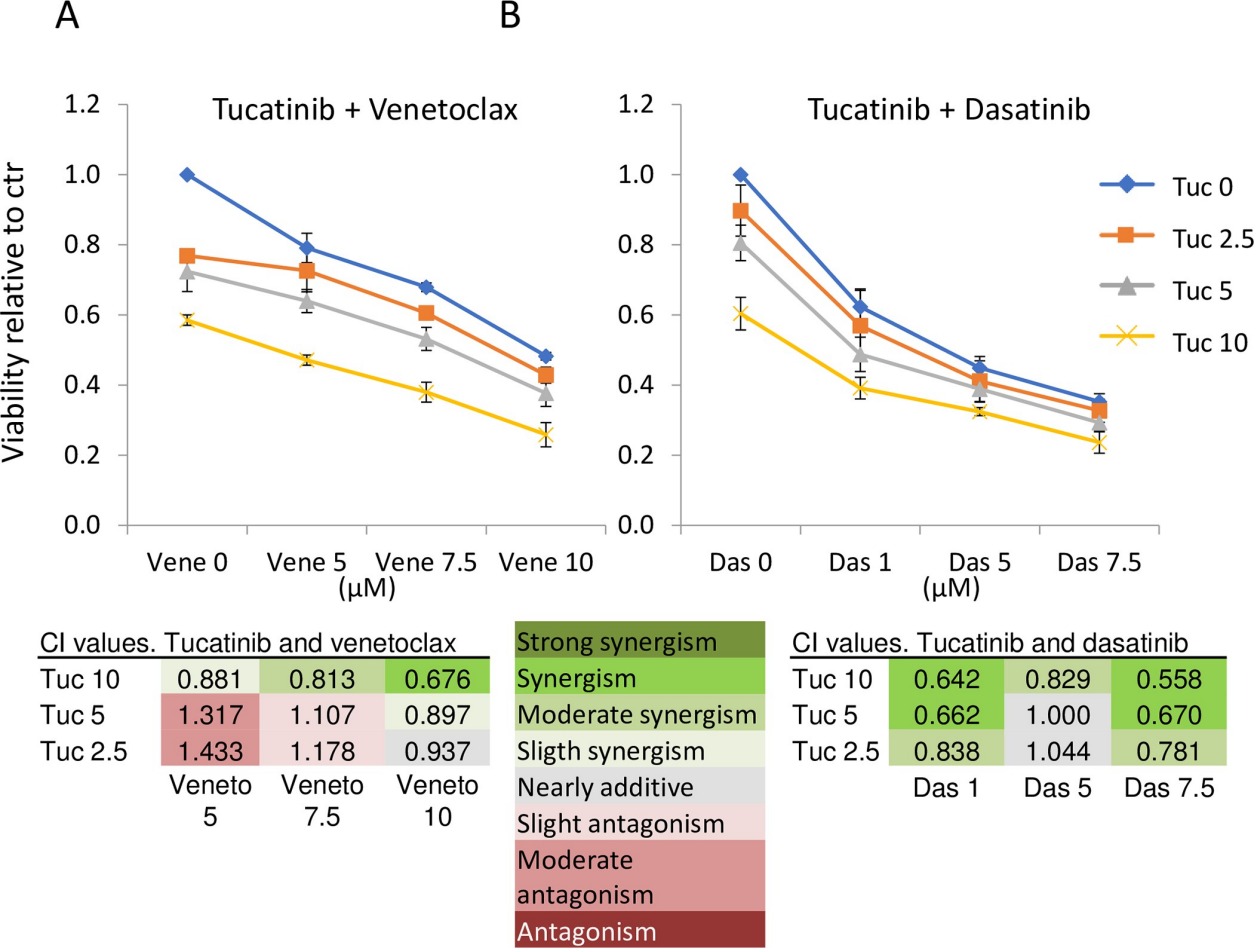
Synergy calculations after combinatorial treatment in KPL4 cells. The cells were treated with different doses of A) tucatinb (0, 2.5, 5, 10 μM) and venetoclax (0,5, 7.5, 10 μM), and B) tucatinib and dasatinib (0,1,5, 7.5 μM) for 72 hours. Error bars represent standard deviation of three biological experiments. Viability values were uploaded to the CalcuSyn software, and combination index values (CI) suggests antagonism, additivity, or synergy. Das = dasatinib, Tuc = tucatinib, Vene = venetoclax.

To include more recently approved and clinically relevant drugs in the study, we wanted to assess the HER2-targeting drug tucatinib and the Bcl-2-inhibitor venetoclax. We also included dasatinib in these experiments. We used KPL4 cells since the effects of dasatinib were the strongest in this cell line (Figs 1A and 2A). Tucatinib, venetoclax and dasatinib all significantly reduced cell viability in a dose-dependent manner (*p* < 0.05, Fig 3). Further, we assessed tucatinib in combination with either venetoclax or dasatinib and explored potential synergy effects using the CalcuSyn software. For the combination of tucatinib and venetoclax, antagonism was detected at the lower drug concentrations, while synergy was detected at higher doses (Fig 4A). For tucatinib and dasatinib, synergy was detected over nearly the entire spectrum of drug concentrations (Fig 4B).

### *In vivo* treatment efficacy using KPL 4 xenografts

**Fig 5:**
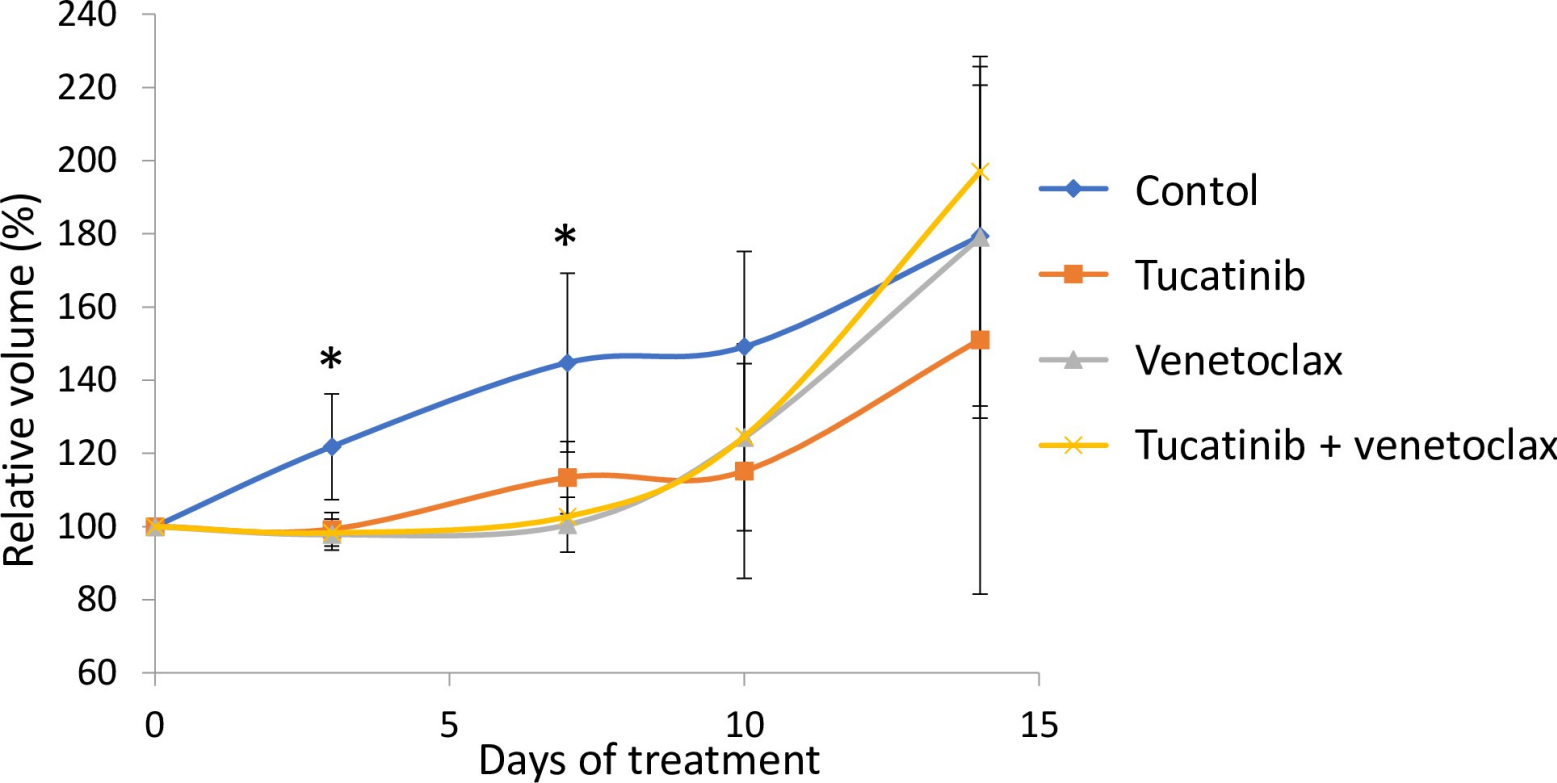
Treatment efficacy of tucatinib and venetoclax *in vivo*. Nude mice with unilateral KPL4 tumors were treated five days per week with tucatinib (25 mg/kg), venetoclax (25 mg/kg), or a combination of the two, or with only vehicle. The figure shows relative tumor volumes (%) compared to volume at the start of treatment. Error bars indicate standard error of the mean, *n* = 6 mice per group. *p < 0.05 between treatment groups and control, Wilcoxon rank-sum test.

Since tucatinib was recently introduced for treatment of HER2+ breast cancers [13], and Bcl-2 is suggested to play a role in this form of cancer [14, 15], we wanted to test tucatinib and venetoclax *in vivo*. Nude mice with KPL4 tumors were treated with tucatinib and venetoclax alone and in combination. There was significant tumor growth inhibition in all treatment groups compared to control at day 3 and 7 (*p* < 0.05, Fig 5), but not at later time points.

**Fig 6:**
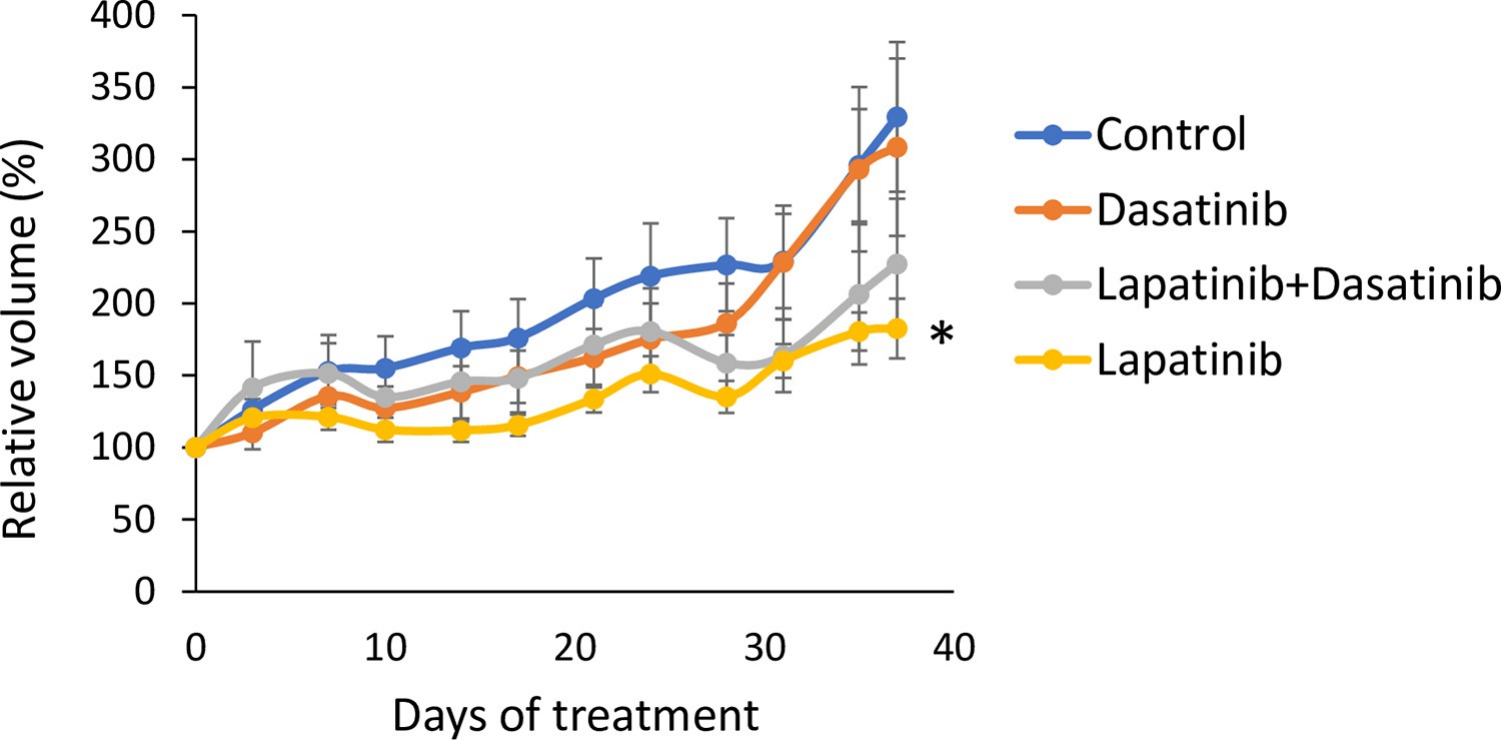
Treatment efficacy of lapatinib and dasatinib *in vivo*. Nude mice with bilateral KPL4 tumors were treated daily with lapatinib (100 mg/kg), dasatinib (10 mg/kg), a combination of both drugs, or vehicle. The figure shows relative tumor volumes (%) compared to volume at the start of treatment. Error bars indicate standard error of the mean, *n* = 6 mice per group. *p < 0.05 between treatment group and control, Wilcoxon rank sum test.

Due to the lack of significant tumor growth inhibition by tucatinib and venetoclax after day 7, the most promising drug combination, dasatinib and lapatinib, from the initial screen and *in vitro* validation was further analyzed. To investigate the combinatorial effect of these drugs, nude mice with KPL4 tumors were treated with lapatinib, dasatinib, or the combination of the two drugs. The effects on tumor growth of dasatinib treatment compared to control was not significant (*p* = 0.651). Monotreatment with lapatinib significantly inhibited tumor growth (*p* = 0.04) on the final day of treatment (Fig 6). Dual treatment with lapatinib and dasatinib showed a tendency of inhibited tumor growth compared to control, although this was not significant (*p* = 0.151). No toxicity was detected in any of the treatment groups.

### Proteomic changes induced by the treatment

**Fig 7:**
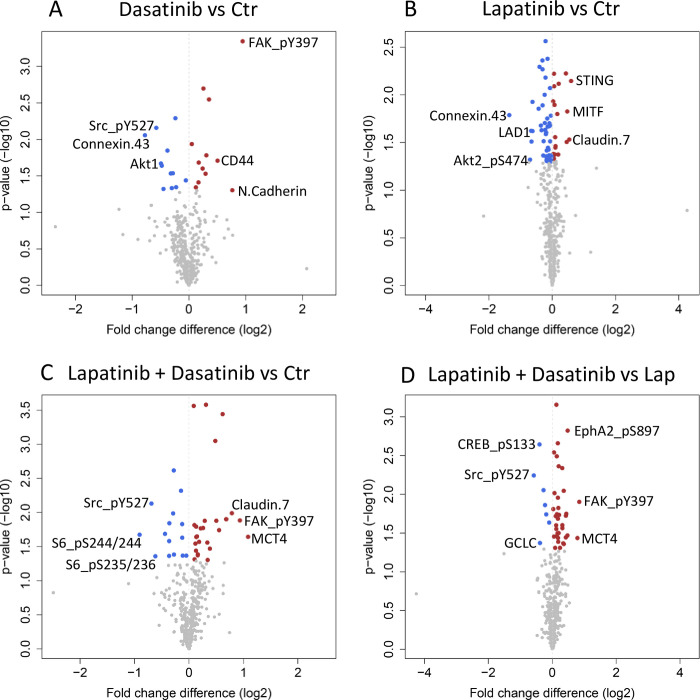
Changes in protein expression levels in tumors treated *in vivo*. Volcano plots of changes in protein levels in tumors treated with A) dasatinib, B) lapatinib, C) lapatinib + dasatinib *vs* control, and D) lapatinib + dasatinib *vs* lapatinib. Colored dots represent proteins significantly (*p* < 0.05) upregulated (red) or downregulated (blue), calculated by Student’s t-test. Proteins with the top three largest fold change in downregulation and upregulation are labelled with names. All significant protein changes are listed in Tables 1 and 2.

To evaluate the changes induced by dasatinib and lapatinib on the protein landscape, tumor tissue from treated mice was harvested and protein lysates were subjected to RPPA analyses. The overall treatment effects on expression levels of all proteins measured (*n* = 417) are presented as a heatmap in S4 Fig. Compared to vehicle treated tumors (control), dasatinib, lapatinib and the drug combination significantly altered the expression of 24, 64 and 41 proteins respectively (*p* < 0.05, Fig 7A–7C and Table 1). In this case, it seems that lapatinib is showing a strong effect on its own, reducing the expression of 53 proteins that were not affected by dasatinib alone or through combinatorial treatment. One protein was downregulated in all treatment groups compared to control, Src_pY527. Enrichment analysis revealed that in all the three treatment groups, HER2-signaling and ERK/MAPK-signaling pathways were among the pathways affected by the treatments (S3 Table).

**Table 1:** Significantly affected proteins by TKIs alone or in combination.

Dasatinib vs Control			Lapatinib vs control			Lapatinib+dasatinib vs control		
Protein	FC	*p*	Protein	FC	*p*	Protein	FC	*p*
4E.BP1	0.24	0.03	14.3.3.zeta	-0.22	0.02	ACVRL1	0.15	0.02
53BP1	-0.24	0.01	Akt2_pS474	-0.70	0.05	Akt2	-0.11	0.04
Akt1	-0.49	0.02	b.Catenin	-0.18	0.05	ATR	0.38	0.03
Caspase.8.cleaved	0.17	0.02	B.Raf	-0.24	0.01	c.Abl_pY412	0.14	0.02
CD4	0.17	0.04	B7.H4	0.09	0.03	C.Raf_pS338	-0.28	<0.01
CD44	0.50	0.02	Bak	0.21	0.01	Calnexin	0.55	0.02
Connexin.43	-0.77	0.01	Bid	-0.02	0.04	Caspase.8.cleaved	0.16	0.04
CREB_pS133	-0.38	0.01	c.IAP2	0.04	0.04	CD20	0.34	0.05
DDR1	-0.28	0.03	c.Jun_pS73	-0.17	0.04	CD4	0.26	0.02
E2F1	0.12	0.05	CD44	-0.34	0.02	CDK1_pT14	-0.28	0.04
FAK_pY397	0.95	<0.01	Cdc6	-0.11	0.05	Claudin.7	0.79	0.01
GRB2	-0.23	0.05	CHD1L	-0.09	0.02	Cox.IV	0.31	<0.01
HES1	-0.48	0.02	CIITA	-0.04	0.02	CREB_pS133	-0.36	0.01
MERIT40_pS29	-0.05	0.04	Claudin.7	0.54	0.03	EphA2_pY588	0.09	<0.01
N.Cadherin	0.76	0.05	COG3	0.08	0.04	EVI1	-0.29	0.01
Oct.4	0.29	0.03	Connexin.43	-1.36	0.02	FAK_pY397	0.93	0.01
Patched	0.05	0.01	Coup.TFII	0.10	0.04	FGF.basic	0.16	0.04
PERK	0.26	<0.01	DAPK2	0.05	0.01	HER2_pY1248	0.68	0.01
PKC.b.II_pS660	-0.30	0.05	DDR1	-0.20	0.03	HES1	-0.44	0.02
S100A4	0.31	0.02	DUSP6	-0.62	0.01	Hif.1.alpha	0.34	0.03
SOX7	0.35	<0.01	EGFR	-0.31	0.01	IDO	0.13	0.02
Src_pY527	-0.57	0.01	eIF4E_pS209	-0.07	0.01	INPP4b	0.48	<0.01
Tuberin	-0.45	0.05	Elk1_pS383	0.16	0.02	IR.b	0.50	0.01
ULK1_pS757	-0.32	0.03	Erk5	-0.15	0.02	IRF.1	-0.12	0.01
			FABP5	-0.31	0.01	MCT4	1.08	0.02
			Folliculin	-0.19	0.02	Mnk1	-0.36	0.04
			Glucocorticoid.Receptor	-0.66	0.03	Myt1	-0.05	0.04
			GRB2	-0.13	0.04	p38.MAPK	-0.12	0.02
			INPP4b	0.43	0.01	PD.1	0.22	0.02
			IR.b	0.46	0.03	PDK1_pS241	-0.15	<0.01
			LAD1	-0.67	0.02	S6_pS235_S236	-0.62	0.04
			MITF	0.47	0.01	S6_pS240_S244	-0.91	0.02
			Mitofusin.2	-0.18	0.05	Smad1	0.10	0.02
			MLKL	-0.27	0.03	SOX7	0.19	0.03
			Mnk1	-0.43	0.01	Src_pY527	-0.69	0.01
			MSI2	0.10	0.03	TFRC	0.62	<0.01
			NDRG1_pT346	-0.62	0.02	TTF1	0.12	0.03
			NF.kB.p65_pS536	0.19	0.04	ULK1_pS757	-0.36	0.03
			Notch1	-0.23	0.02	Wee1_pS642	0.10	0.05
			P.Cadherin	-0.04	0.05	X53BP1	0.13	0.04
			p53	0.07	0.01	ZEB1	0.29	0.01
			Patched	0.06	0.01			
			PDHA1	-0.16	0.05			
			PDHK1	0.03	0.01			
			PDK1_pS241	-0.15	<0.01			
			PHLPP	-0.04	0.04			
			PIP4K2A	-0.10	0.04			
			PKM2	-0.16	0.02			
			PLC.gamma1	-0.10	0.02			
			PR	-0.17	0.05			
			PTPN12	-0.19	0.04			
			RIP3	0.05	0.05			
			RSK1	-0.07	0.02			
			SGK1	-0.36	0.02			
			SHP.2_pY542	-0.21	<0.01			
			SIRP.alpha	-0.28	0.04			
			SLC1A5	-0.40	0.01			
			Smad4	-0.03	0.05			
			Src_pY527	-0.12	0.04			
			STING	0.60	0.01			
			VHL	-0.21	0.01			
			XPF	-0.07	0.03			
			YAP_pS127	-0.31	<0.01			
			YES1	-0.13	0.04			

Log_2_-fold change values (FC) and *p*-values (*p*) for significant protein level alterations in different treatment groups from *in vivo* experiments (*p* < 0.05; Student’s t-test). Dasatinib *vs* control: *n* = 24. Lapatinib *vs* control: *n* = 64. Lapatinib+dasatinib *vs* control: *n* = 41.

**Fig 8:**
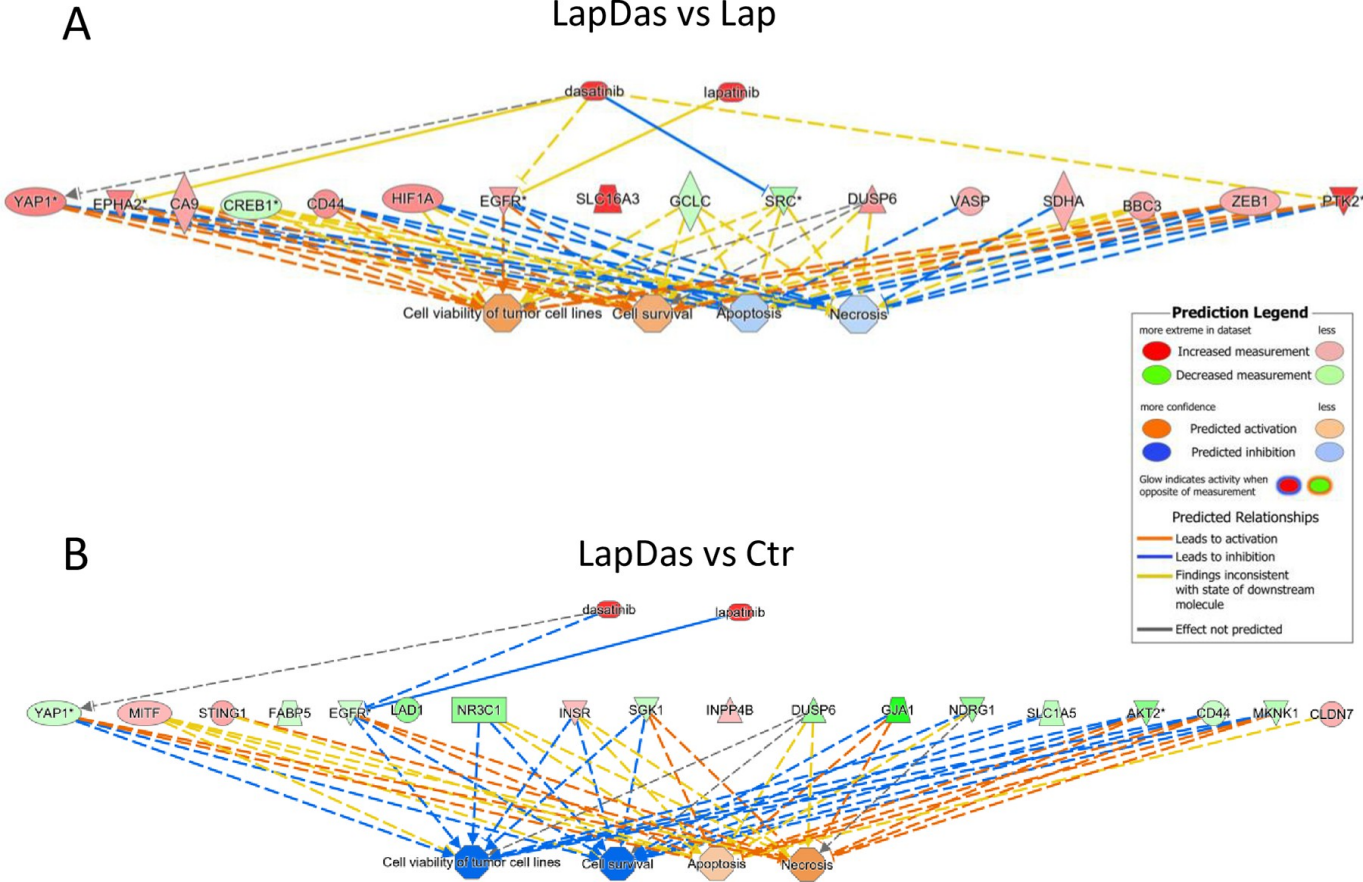
Predicted effects on protein expression upon treatment. The IPA core analyses and predictions of downstream regulations of the proteins measured in tumors treated with lapatinib and dasatinib compared to A) lapatinib alone, and B) control tumors. The predicted effects of the changes in protein expression in lapatinib + dasatinib (LapDas) *vs* lapatinib (Lap) show activation of cell viability and survival, and inhibition of apoptosis and necrosis (A). This is the opposite of what is predicted when comparing the changes in protein expression in LapDas *vs* control (Ctr) tumors (B). The prediction legend explains the color codes of the predicted inhibition and activation. For visualization purposes, only proteins with an |FC| > 0.3 and *p*-value < 0.05 are shown. All significantly regulated proteins are listed in Tables 1 and 2.

Since lapatinib alone inhibited tumor growth, while the addition of dasatinib did not influence this, we evaluated the protein levels that were significantly changed between combinatorial treatment and lapatinib alone, i.e., the additional effect of dasatinib. This resulted in 43 proteins being significantly altered (Fig 7D, Table 2). According to enrichment analysis, cell viability and cell survival were predicted to be increased with a positive Z-score, while apoptosis and necrosis were predicted to be decreased with a negative Z-score (Fig 8A). The opposite effect was obtained when comparing combinatorial treatment with control or each monotreatment with control (Fig 8B, S4 Table). This suggests that the addition of dasatinib to lapatinib may cause changes in the protein expression having antagonistic effects, further explaining why the drug combination did not enhance tumor growth inhibition compared to lapatinib alone.

**Table 2:** Significantly affected proteins by lapatinib and dasatinib in combination vs lapatinib alone in HER2+ tumors.

Protein	FC	*p*
Akt2	-0.10	0.02
B7.H3	0.18	0.02
c.Abl_pY412	0.15	0.02
c.Jun_pS73	0.18	0.04
C.Raf_pS338	-0.19	0.02
CA9	0.34	0.04
Caspase.3.cleaved	0.13	<0.01
Caspase.8.cleaved	0.18	0.01
CD44	0.43	0.02
CENP.A	0.20	0.03
CHD1L	0.13	0.03
CIITA	0.05	<0.01
CREB_pS133	-0.40	<0.01
DUSP6	0.42	0.04
EGFR	0.36	0.01
EphA2_pS897	0.48	<0.01
FAK_pY397	0.85	0.01
GCLC	-0.39	0.04
GCLM	0.19	0.04
Hif.1.alpha	0.44	0.02
IRF.1	-0.23	0.01
MCT4	0.79	0.04
MelanA	0.03	0.02
PHLPP	0.09	0.05
PKCa	0.17	0.03
PRC1_pT481	0.14	0.03
Puma	0.36	0.04
SDHA	0.31	<0.01
SHP.2_pY542	0.17	<0.01
Slfn11.G.C	0.20	<0.01
Smad1	0.19	0.01
Sox17	0.07	0.01
Src_pY527	-0.57	0.01
TRIM24	0.24	0.05
TTF1	0.11	0.02
ULK1_pS757	-0.27	0.01
VASP	0.32	0.03
VHL	0.21	0.02
Wee1_pS642	0.14	<0.01
X14.3.3.zeta	0.12	0.03
XPF	0.05	0.04
YAP_pS127	0.48	0.03
ZEB1	0.32	0.03

Log_2_-fold change values (FC) and *p*-values (*p*) for significant protein level alterations in different treatment groups from *in vivo* experiments (*p* < 0.05). Lapatinib+dasatinib *vs* lapatinib: *n* = 43.

## Discussion

Despite continuous improvement of HER2-targeted breast cancer therapy over the past decades, there is still a need for new strategies for patients that respond poorly to treatment. High-throughput drug screens to identify promising novel combinations is a powerful method to evaluate a large number of possibilities in an efficient way. There are numerous FDA-approved and clinically tested drugs with an acceptable safety profile that are used for other indications than breast cancer. In this study, we have screened 278 drugs alone and in combination with lapatinib and/or trastuzumab in HER2+ breast cancer cells with the aim of identifying combinations that are efficacious in poorly responding cells. By evaluating already approved therapeutics or drugs tested in patient trials, clinical translation may be easier than for exploration of novel and unapproved drugs.

The drug screen revealed an overall stronger combinatorial effect in cells treated with both trastuzumab and lapatinib compared to each drug independently (Fig 1). We selected nine drugs that ranked among top ten in the ability to reduce cell viability in combinatorial treatment with either lapatinib or trastuzumab, or in combination with both. The *in vitro* validations of the nine selected drugs showed little or no improved growth inhibitory effect by adding trastuzumab to lapatinib (Fig 2, S3 Fig). We speculate that the lack of improved growth inhibition by adding trastuzumab may be explained by activating mutations in PIK3CA and through the loss of *PTEN* in both KPL4 and SUM190PT cells [6]. These mutations have been shown to contribute to resistance towards trastuzumab [16–18] and lapatinib [19] in HER2+ breast cancer. In addition, several studies have demonstrated that there is a complex interplay of HER2 and other proteins that impact the downstream signaling in HER2 positive tumors which can result in trastuzumab resistance [20–22]. This emphasizes that the results from the high-throughput screen should be validated in additional cell lines.

From previous work, we know that the growth inhibitory effects of lapatinib and trastuzumab in KPL4 and SUM190PT cells are limited [6, 23]. As is commonly known, lapatinib is not frequently used as first line treatment in HER2+ breast cancers, although it is recommended as a treatment option at later stages of the disease [24]. To investigate more contemporary drugs, the TKI tucatinib was included as an updated alternative to lapatinib. Lapatinib inhibits both EGFR and HER2, while tucatinib has a strong specificity for HER2 [25], which is reported to give fewer side effects in clinical use [26]. In addition, navitoclax was replaced with venetoclax based on fewer adverse side effects due to its high Bcl-x_L_ specificity [27]. For future studies, it would be interesting to investigate more novel drugs, such as the monoclonal antibody pertuzumab and the antibody-drug conjugates T-DM1 and/or trastuzumab deruxtecan, which recently has been approved as standard treatment for early and advanced HER2+ breast cancer.

Venetoclax was approved in 2016 by the FDA for use in chronic lymphocytic leukemia patients [28]. The effects of venetoclax has been clinically tested in ER-positive, HER2-negative metastatic breast cancer patients (NCT03584009) in combination with fulvestrant, and was found to be inferior to endocrine treatment alone [29]. A clinical trial combining venetoclax and trastuzumab-emtansine in HER2+ breast cancer patients was initiated but was subsequently terminated due to strategic prioritization (NCT04298918).

We demonstrated inhibition of tumor growth by tucatinib and venetoclax *in vivo* compared to control after the first week of treatment (Fig 5). Furthermore, there was a tendency towards inferior growth inhibition in the combination treatment compared to the monotreatment, which could mirror the antagonistic effect detected *in vitro* (Fig 4A). For future work, higher doses could be tested *in vivo*. Floros et al. explored inhibition of Bcl-2 in combination with lapatinib in HER2+ breast cancer cell lines. They did not detect a sensitizing effect of venetoclax to lapatinib in BT-474 or MDA-MB-453 cell lines [30]. In contrast to the KPL4 (and SUM190PT) cells, BT-474 is ER-positive, and MDA-MB-453 cells are *PTEN* wildtype [6]. These features suggest that the cells rely on different signaling pathways, which may explain the discrepancy in response. In a broader sense, it highlights cell line specificity and the need for reliable biomarkers to identify patients that could benefit from a particular treatment.

The drug screen identified multiple interesting drugs for further investigations (S2 Table), and dasatinib was one of the candidates. Overexpression of Src has been observed together with EGFR and HER2-overexpression [31, 32], which supports the concept that dual Src- and EGFR-/HER2-targeting may work synergistically in HER2+ cells. The *in vitro* validation of dasatinib in KPL4 cells further argues for the value of Src-inhibition, as the drug demonstrated growth inhibiting effects in combination with lapatinib. This is in line with results from a study by Stanley et al., detecting synergistic effects when combining lapatinib and dasatinib in HER2+ breast cancer cell lines [33]. On the other hand, antagonism was also reported in other cell lines, which could be linked to the intrinsic HER2- and Src-expression levels in the cells [33]. KPL4 cells express both [34], which may explain why lapatinib and dasatinib inhibited cell growth *in vitro* in the study presented here.

Dasatinib is a multi-target kinase inhibitor affecting several protein kinases, including Bcr-Abl and Src family kinases [35, 36]. The drug was approved by the FDA in 2006 [37], and is currently used for treating Philadelphia-chromosome positive chronic myeloid leukemia patients. Src signaling is involved in oncogenic processes and mediates downstream signaling from receptors such as EGFR and HER2 [31]. The use of dasatinib treatment in breast cancer has been pre-clinically and clinically tested for both triple-negative (as monotherapy or with chemotherapy) [38, 39] and HER2+ breast cancer (as monotherapy or with trastuzumab and paclitaxel) [40, 41]. The clinical studies do not conclude that the efficacy is sufficient to implement dasatinib but suggest an unexplored potential for including the drug in combinatorial treatments of metastatic breast cancer.

Although dasatinib displays no significant tumor growth inhibiting effects *in vivo* (Fig 6), 24 protein levels were significantly altered in the dasatinib-treated tumors compared to control tumors. When looking into these 24 proteins, two of the proteins are direct targets of dasatinib: DDR1 [42] and phosphorylated Src (Src_pY527) [43] (S5 Fig). Overall, Src_pY527 was one of the most downregulated proteins in tumors treated with dasatinib. Src plays a role in cellular responses to extra- and intracellular signals and is known as a family of kinases with oncogenic potential. The strong downregulation of this protein describes one of the anti-tumorigenic changes induced by the treatment and shows that the given dose of dasatinib was sufficiently high to reach the tumors and regulate one of its main downstream targets. However, despite induction of molecular changes, the effects were not substantial enough to significantly inhibit tumor growth. This discrepancy may in part be explained by the balance between pro-tumorigenic and anti-tumorigenic protein regulations and the redundancy in tumor growth regulation. The *in silico* analyses predicted inhibited apoptosis and enhanced cell viability in tumors treated with the combination compared to lapatinib alone (Fig 8). Finally, the tumor microenvironment in the animal model may impact the results and explain why we do not observe the same growth inhibition *in vivo* as *in vitro*.

## Conclusion

We investigated novel treatment combinations for HER2+ breast cancers that respond poorly to trastuzumab and lapatinib. Combinatorial effect of dasatinib and lapatinib showed potential *in vitro*, but this could not be validated *in vivo* where molecular analyses suggested a proteomic change towards anti-apoptotic effect. The results demonstrate the importance of combining *in vivo* response data with subsequent molecular analyses to unravel possible treatment combinations for tumors not responding to standard treatment.

## Supporting information

S1 Fig A schematic overview of the drug screen setup. (PDF)

S2 Fig Viability curves from high-throughput screen. Relative viability of selected drugs from the high-throughput screen in increasing doses alone and in combination with lapatinib (0,1 μM) and trastuzumab (10 μg/ml) in A) KPL4 and B) SUM190PT cells.
(PDF)

S3 Fig *In vitro* validation of targets from screen. Treatment with increasing doses of the drugs alone and in combination with trastuzumab (10 μg/mL) and lapatinib (0.1 μM) in A) KPL4 cells, and B) SUM190PT cells. C) Table of *p*-values. Student’s t-test *p*-values calculated between treatment groups at the indicated concentrations. Error bars represent standard deviation of 4 technical replicates repeated in two biological replicates.
(PDF)

S4 Fig Heatmap of protein measurements. Protein data from tumors treated in vivo with vehicle (Ctr), lapatinib, dasatinib or lapatinib+dasatinib (LapDas) presented in a heatmap sorted on treatment and clustered using pearson average distances on protein expression.
(PDF)

S5 Fig Protein expression of DDR1 and Src_pY527. Protein data from tumors treated with vehicle, lapatinib, dasatinib or lapatinib+dasatinib (LapDas). *p < 0.05, **p < 0.01, Student’s t-test compared to control. Error bars represent standard deviation of protein measurements from three tumors per treatment group.
(PDF)

S1 Table List of drugs from high-throughput screen. (PDF)

S2 Table *In vitro* validated drugs and their targets. (PDF)

S3 Table IPA pathways. (PDF)

S4 Table IPA diseases and functions. (PDF)

## Abbreviations

AUC: area under curve
CTG: CellTiter-Glo
DMSO: dimetyl sulfoxide
DSS: drug sensitivity score
FC: fold change
FDA: The US Food and Drug Administration
HER2: Human epidermal growth factor
RPPA: Reverse Phase Protein Array
TKI: Tyrosine kinase inhibitor
