# Supplementary figures and images for: High-throughput screen in vitro identifies dasatinib as a candidate for combinatorial treatment with HER2-targeting drugs in breast cancer
Source: PLoS One. 2023 Jan 27;18(1):e0280507. doi: 10.1371/journal.pone.0280507 (PMC9882887; doi:10.1371/journal.pone.0280507)

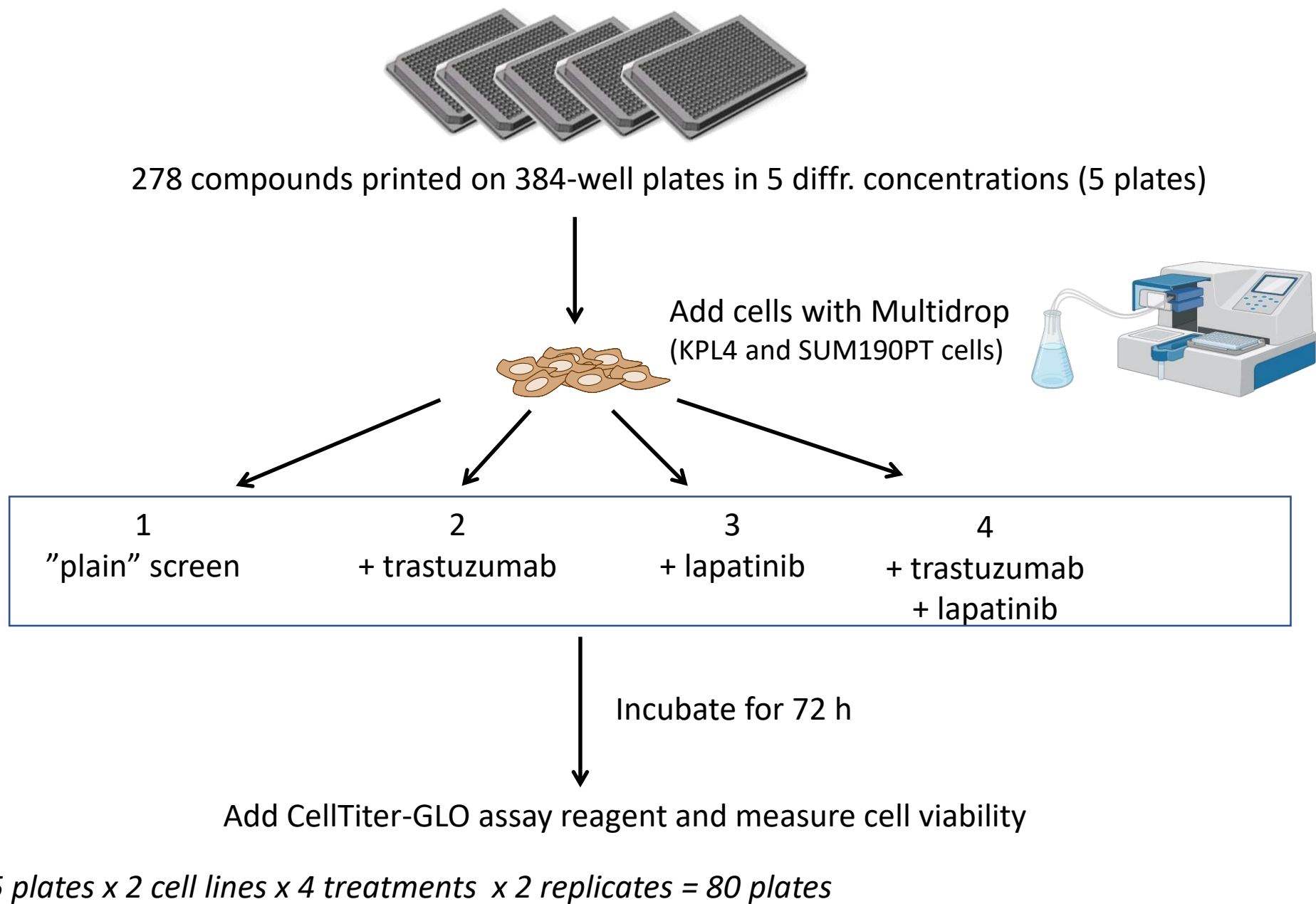

**S1 Fig. A schematic overview of the drug screen setup.** Partially created with BioRender.com.

Supplement: S1 Fig — (PDF) [file pone.0280507.s001.pdf]
