# Supplementary material for: High-throughput screen in vitro identifies dasatinib as a candidate for combinatorial treatment with HER2-targeting drugs in breast cancer
Source: PLoS One. 2023 Jan 27;18(1):e0280507. doi: 10.1371/journal.pone.0280507 (PMC9882887; doi:10.1371/journal.pone.0280507)
Supplement: S2 Fig — Relative viability of selected drugs from the high-throughput screen in increasing doses alone and in combination with lapatinib (0,1 μM) and trastuzumab (10 μg/ml) in A) KPL4 and B) SUM190PT cells. (PDF) [file pone.0280507.s002.pdf]

KPL4

Drug	EC50	drug alone	EC50	drug + tras (10µg/mL)	EC50	drug + lap (100 nM)	EC50	drug + tras + lap
**Carboplatin**	10000	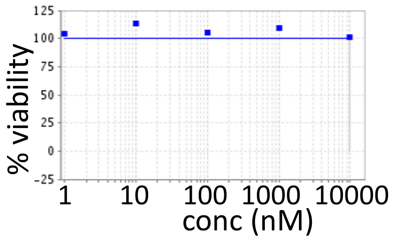	10000	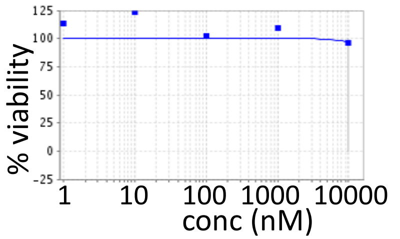	10000	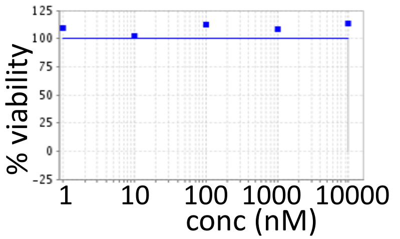	10000	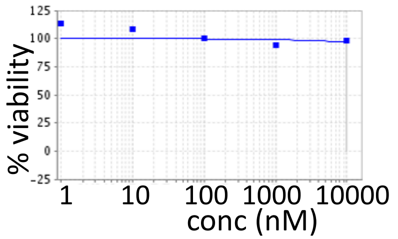
**CUDC-101**	1447	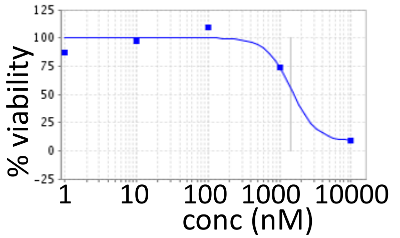	1743	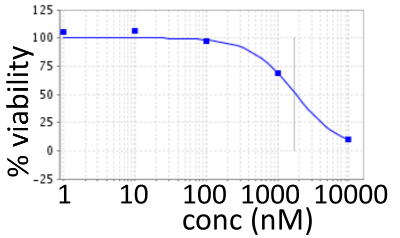	1110	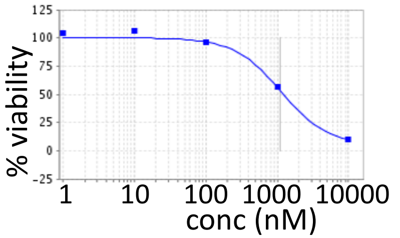	579	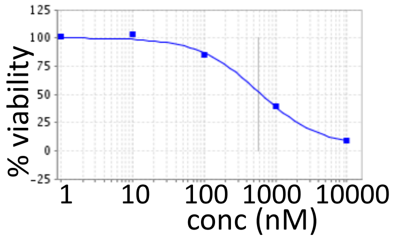
**Danusertib**	10000	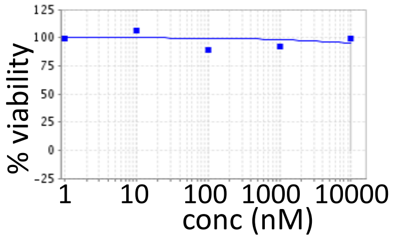	56	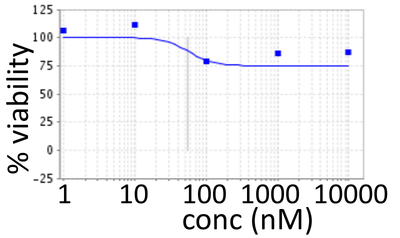	50	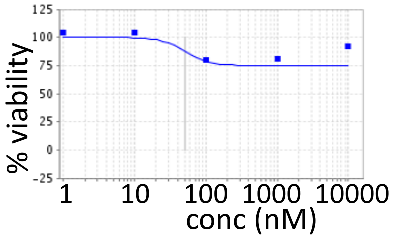	74	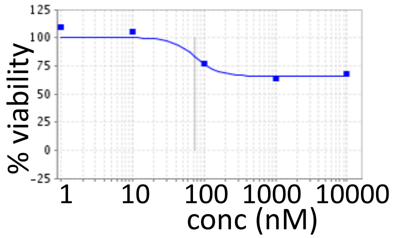

Supplementary Figure S2: Viability curves of selected drugs from high-throughput screen in KPL4 cells. Y-axis: Viability (%), X-axis: drug conc (nM)entration. Drugs are sorted alphabetically. Half maximal effective conc (nM)entrations (EC50) are presented to the left of each curve.

KPL4 continued

Drug	EC50	drug alone	EC50	drug + tras (10µg/mL)	EC50	drug + lap (100 nM)	EC50	drug + tras + lap
**Dasatinib**	1000	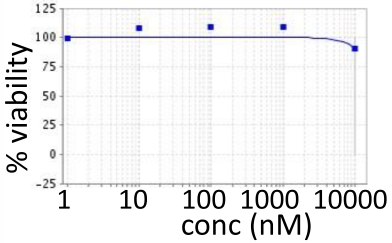	565	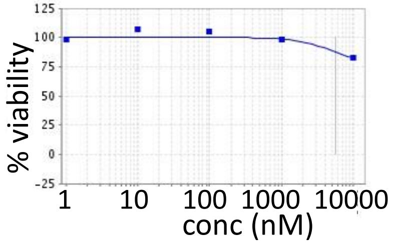	1000	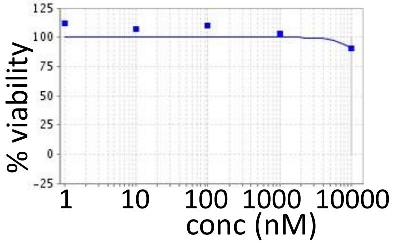	68	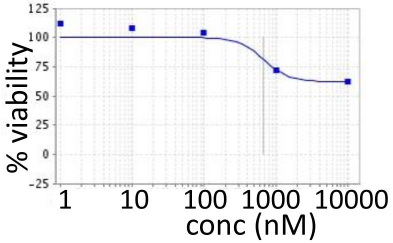
**GDC-0941**	188	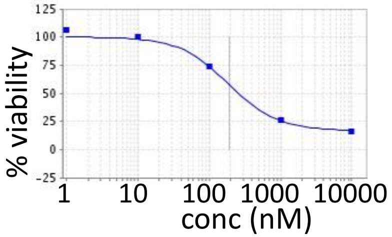	179	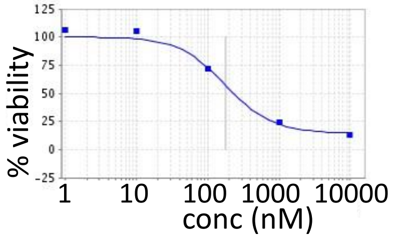	118	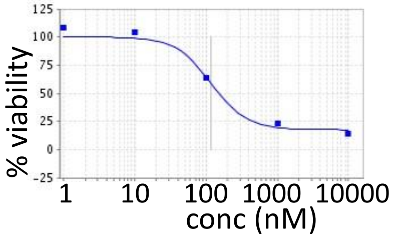	121	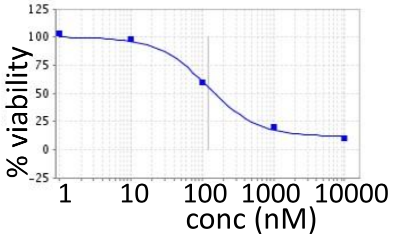
**Navitoclax**	1612	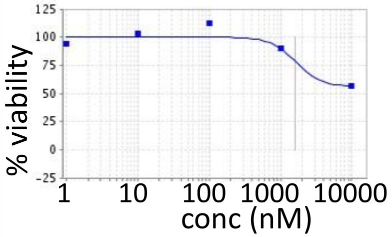	7200	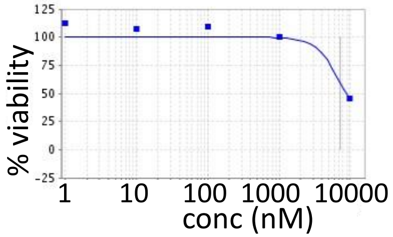	1719	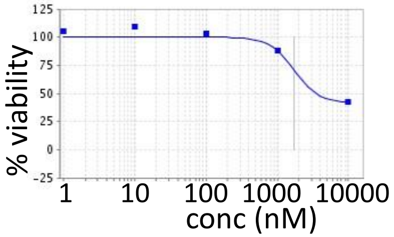	2288	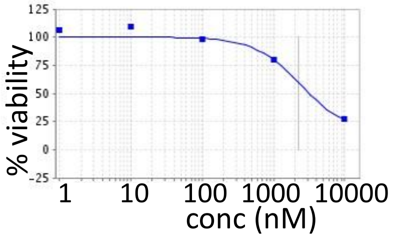

Supplementary Figure S2: Viability curves of selected drugs from high-throughput screen in KPL4 cells. Y-axis: Viability (%), X-axis: drug conc (nM)entration. Drugs are sorted alphabetically. Half maximal effective conc (nM)entrations (EC50) are presented to the left of each curve.

KPL4 continued

Drug	EC50	drug alone	EC50	drug + tras (10µg/mL)	EC50	drug + lap (100 nM)	EC50	drug + tras + lap
**Prednisone**	10000	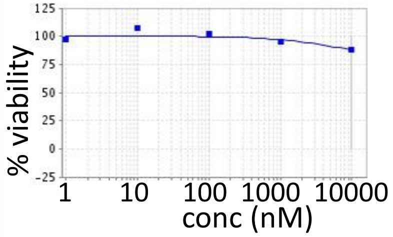	3656	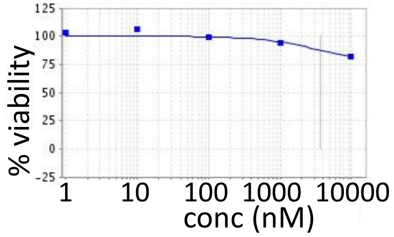	10000	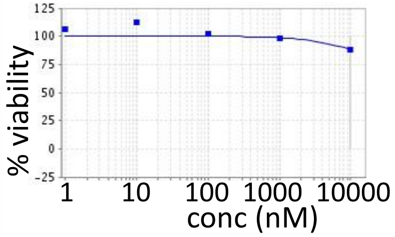	5902	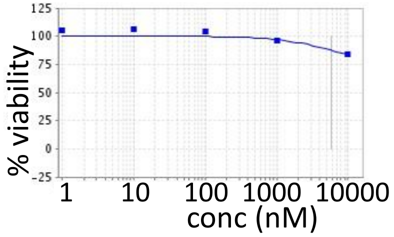
**OSI-906**	10000	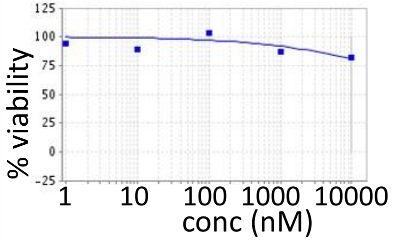	10000	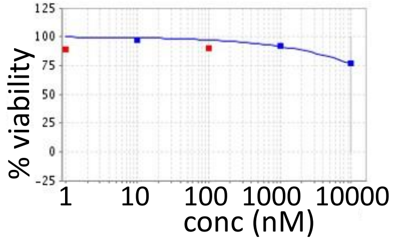	1647	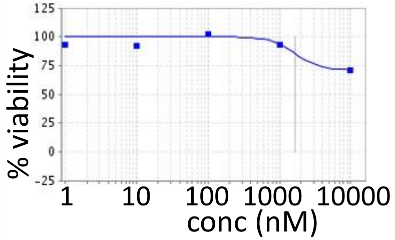	5315	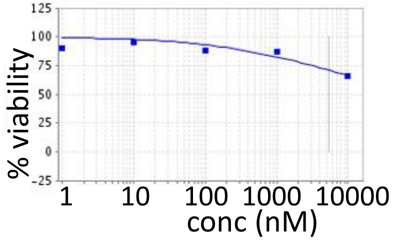
**Quisinostat**	58	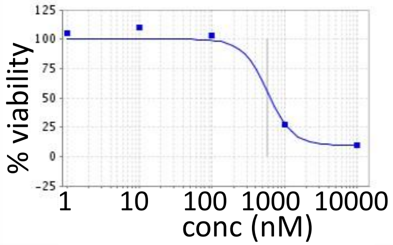	46	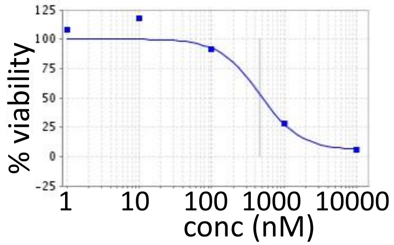	35	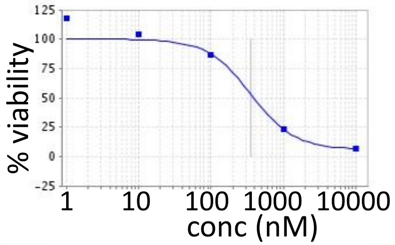	23	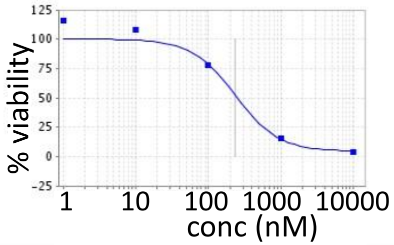

Supplementary Figure S2: Viability curves of selected drugs from high-throughput screen in KPL4 cells. Y-axis: Viability (%), X-axis: drug conc (nM)entration. Drugs are sorted alphabetically. Half maximal effective conc (nM)entrations (EC50) are presented to the left of each curve.

SUM190PT

Drug	EC50	drug alone	EC50	drug + tras (10μg/mL)	EC50	drug + lap (100 nM)	EC50	drug + tras + lap
**Carboplatin**	10000	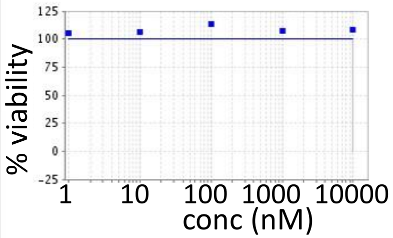	10000	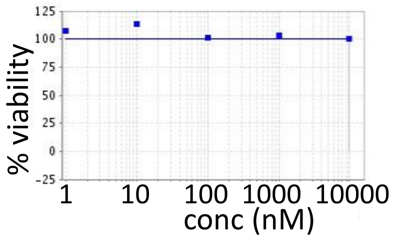	10000	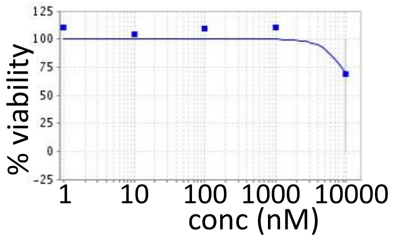	10000	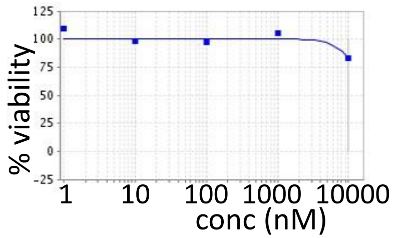
**CUDC-101**	269	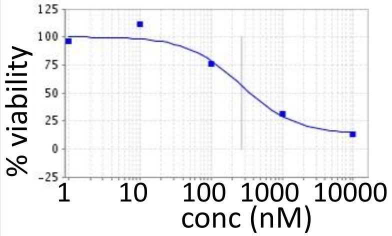	299	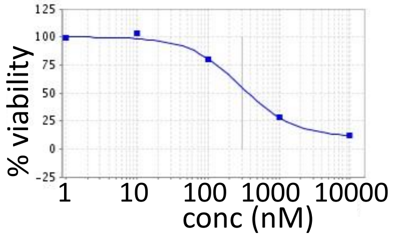	327	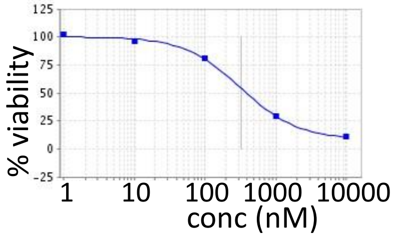	196	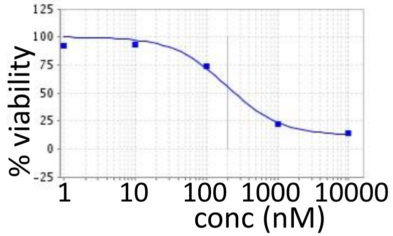
**Danusertib**	1429	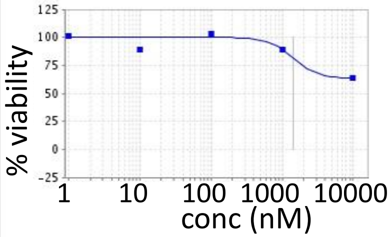	935	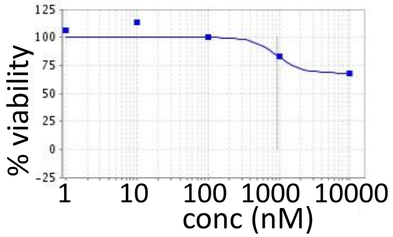	298	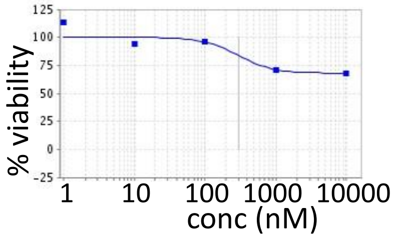	6552	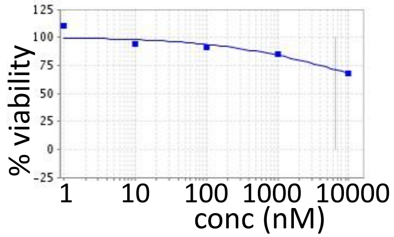

Supplementary Figure S2: Viability curves of selected drugs from high-throughput screen in SUM190PT cells. Y-axis: Viability (%), X-axis: drug conc (nM)entration. Drugs are sorted alphabetically. Half maximal effective conc (nM)entrations (EC50) are presented to the left of each curve.

SUM190PT continued

Drug	EC50	drug alone	EC50	drug + tras (10µg/mL)	EC50	drug + lap (100 nM)	EC50	drug + tras + lap
**Dasatinib**	1000	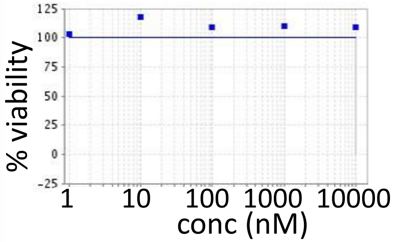	1000	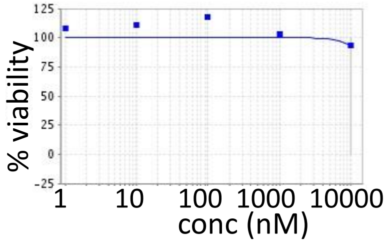	1000	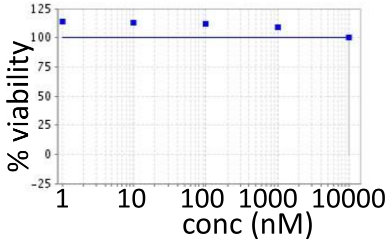	1000	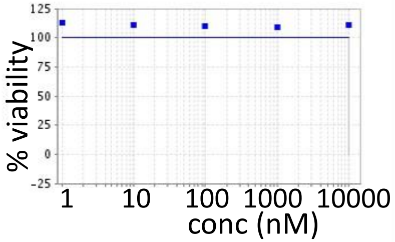
**GDC-0941**	556	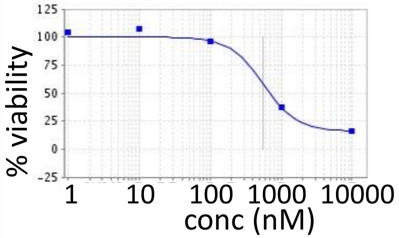	377	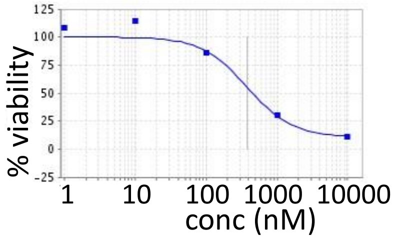	236	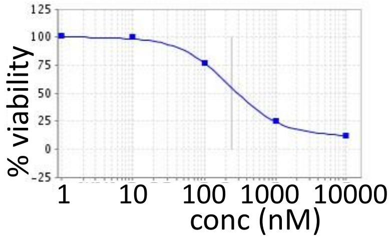	147	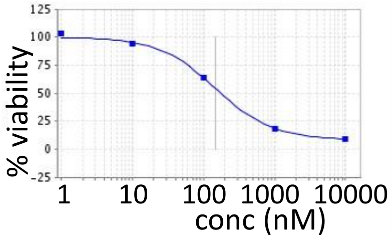
**Navitoclax**	3404	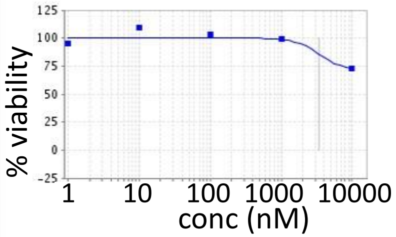	828	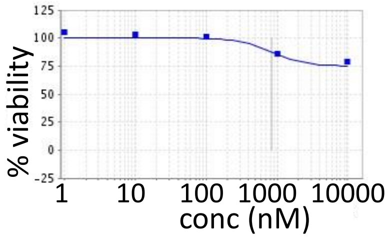	2472	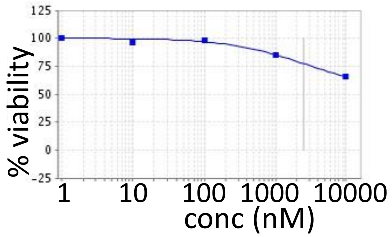	3494	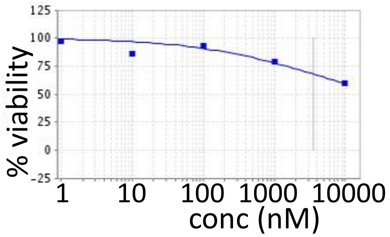

Supplementary Figure S2: Viability curves of selected drugs from high-throughput screen in SUM190PT cells. Y-axis: Viability (%), X-axis: drug conc (nM)entration. Drugs are sorted alphabetically. Half maximal effective conc (nM)entrations (EC50) are presented to the left of each curve.

SUM190PT continued

Drug	EC50	drug alone	EC50	drug + tras (10µg/mL)	EC50	drug + lap (100 nM)	EC50	drug + tras + lap
**Prednisone**	10000	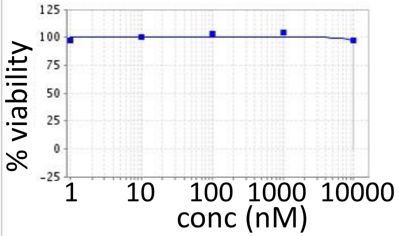	10000	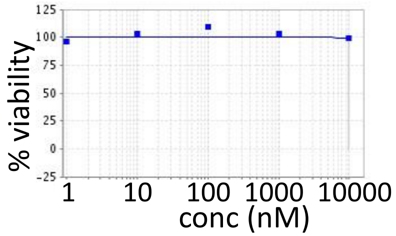	10000	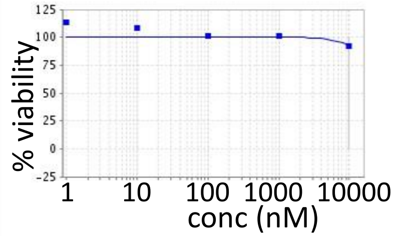	10000	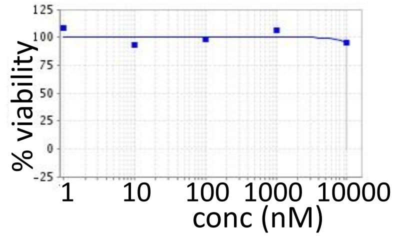
**OSI-906**	1096	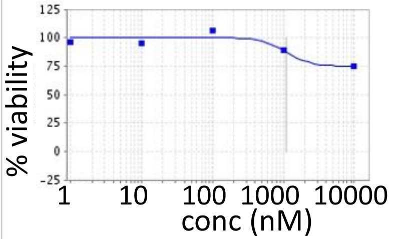	1943	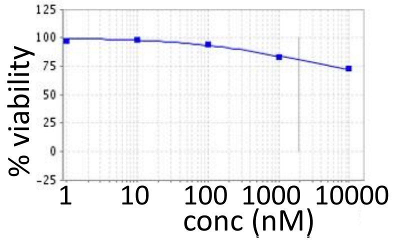	10000	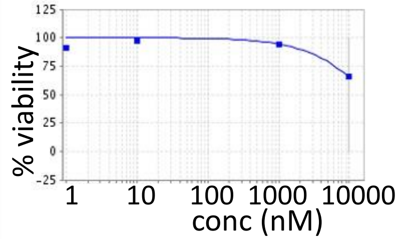	10000	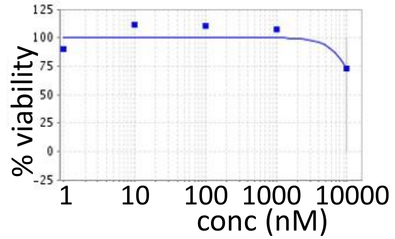
**Quisinostat**	63	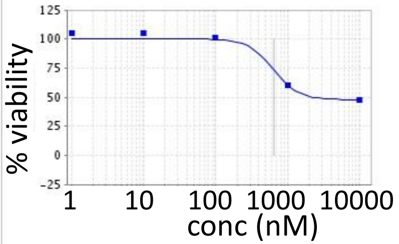	60	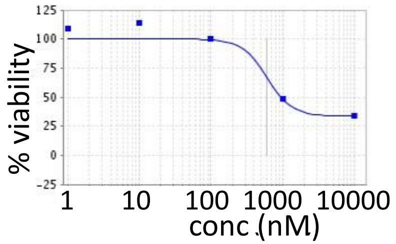	49	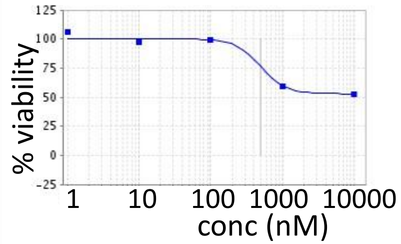	47	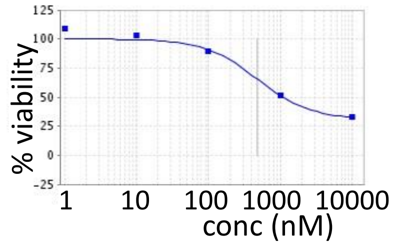

Supplementary Figure S2: Viability curves of selected drugs from high-throughput screen in SUM190PT cells. Y-axis: Viability (%), X-axis: drug conc (nM)entration. Drugs are sorted alphabetically. Half maximal effective conc (nM)entrations (EC50) are presented to the left of each curve.
