# Supplementary material for: High-throughput screen in vitro identifies dasatinib as a candidate for combinatorial treatment with HER2-targeting drugs in breast cancer
Source: PLoS One. 2023 Jan 27;18(1):e0280507. doi: 10.1371/journal.pone.0280507 (PMC9882887; doi:10.1371/journal.pone.0280507)
Supplement: S3 Fig — Treatment with increasing doses of the drugs alone and in combination with trastuzumab (10 μg/mL) and lapatinib (0.1 μM) in A) KPL4 cells, and B) SUM190PT cells. C) Table of p-values. Student’s t-test p-values calculated between treatment groups at the indicated concentrations. Error bars represent standard deviation of 4 technical replicates repeated in two biological replicates. (PDF) [file pone.0280507.s003.pdf]

S3 Fig.

Carboplatin

A

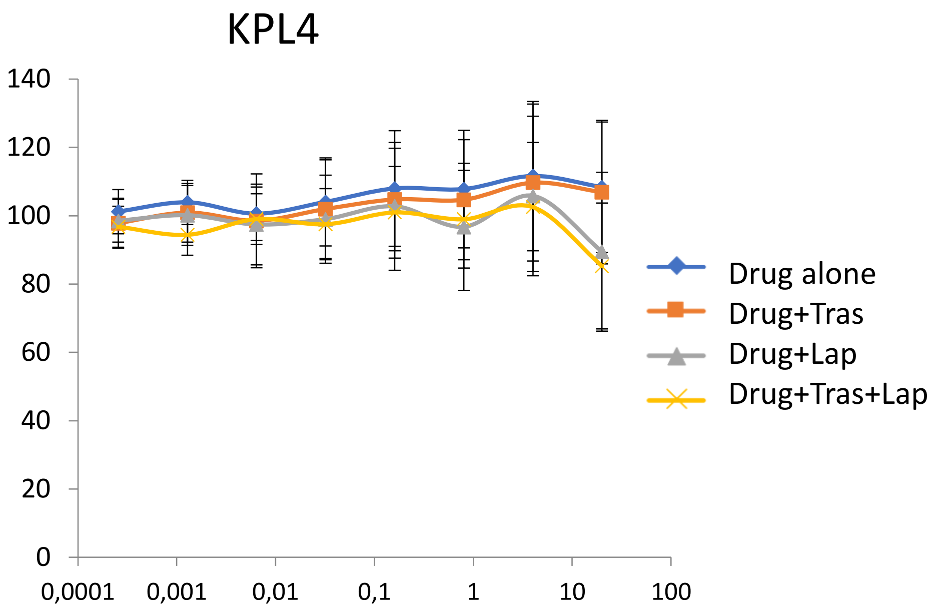

B

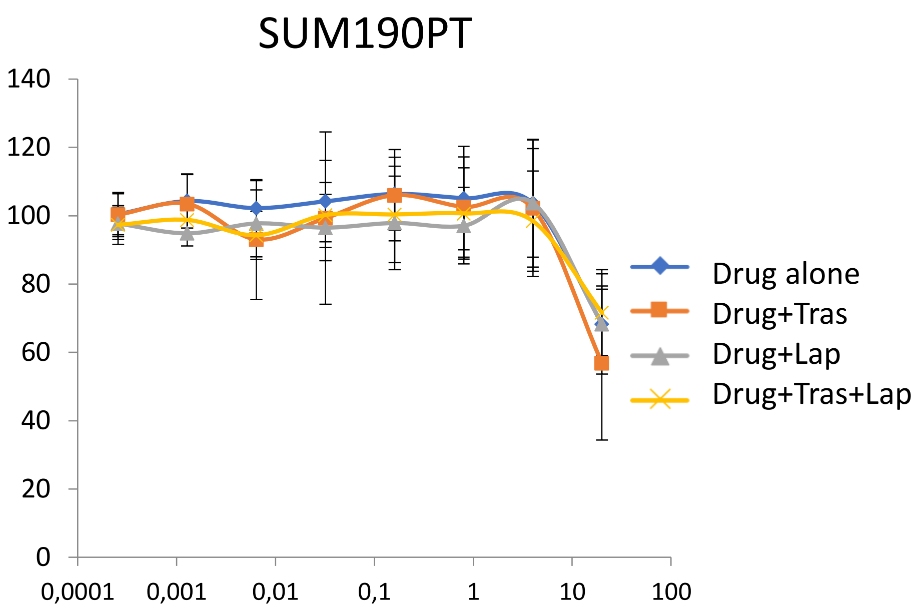

C

Student's t-test *p*-values

	**KPL4**			**SUM190PT**		
Conc (µM)	Carbo vs Carbo+Tras	Carbo vs Carbo+Lap	Carbo vs Carbo+Tras+Lap	Carbo vs Carbo+Tras	Carbo vs Carbo+Lap	Carbo vs Carbo+Tras+Lap
0.000256	0.34	0.41	0.18	0.97	0.33	0.30
0.00128	0.44	0.34	0.01	0.84	0.01	0.12
0.0064	0.72	0.54	0.69	0.20	0.33	0.05
0.032	0.76	0.44	0.29	0.62	0.18	0.46
0.16	0.70	0.57	0.38	0.95	0.19	0.35
0.8	0.73	0.24	0.28	0.74	0.25	0.54
4	0.87	0.62	0.39	0.88	0.99	0.54
20	0.89	0.10	0.03	0.25	0.99	0.63

S3 Fig. In vitro validation of targets from screen. Treatment with increasing doses of the drugs alone and in combination with trastuzumab (10 µg/mL) and lapatinib (0.1 µM) in A) KPL4 cells, and B) SUM190PT cells. C) Table of p-values. Student's t-test p-values calculated between treatment groups at the indicated concentrations. Error bars represent standard deviation of 4 technical replicates repeated in two biological replicates.

S3 Fig.

CUDC-101

A

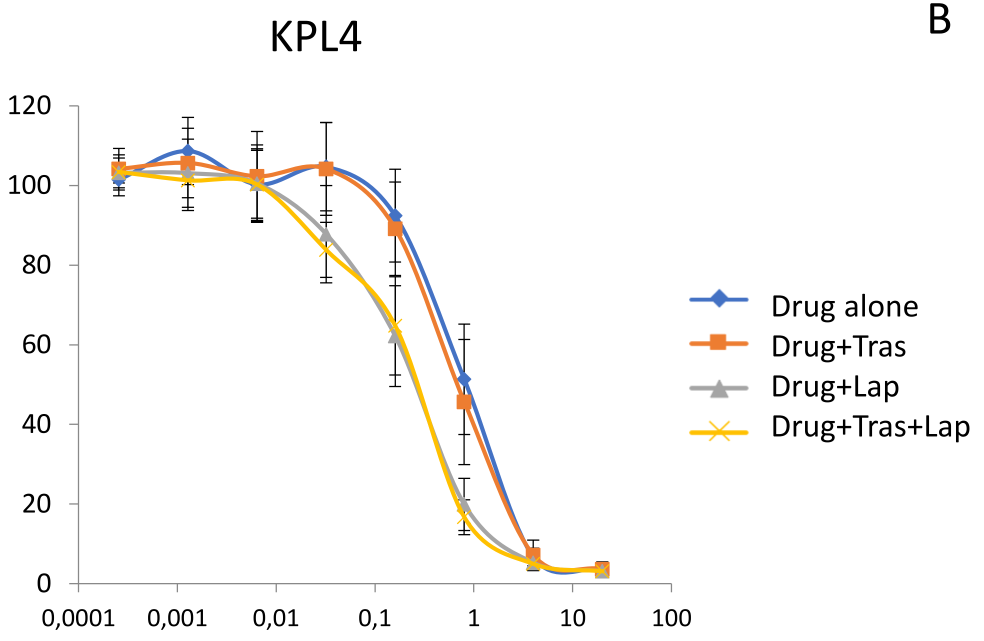

B

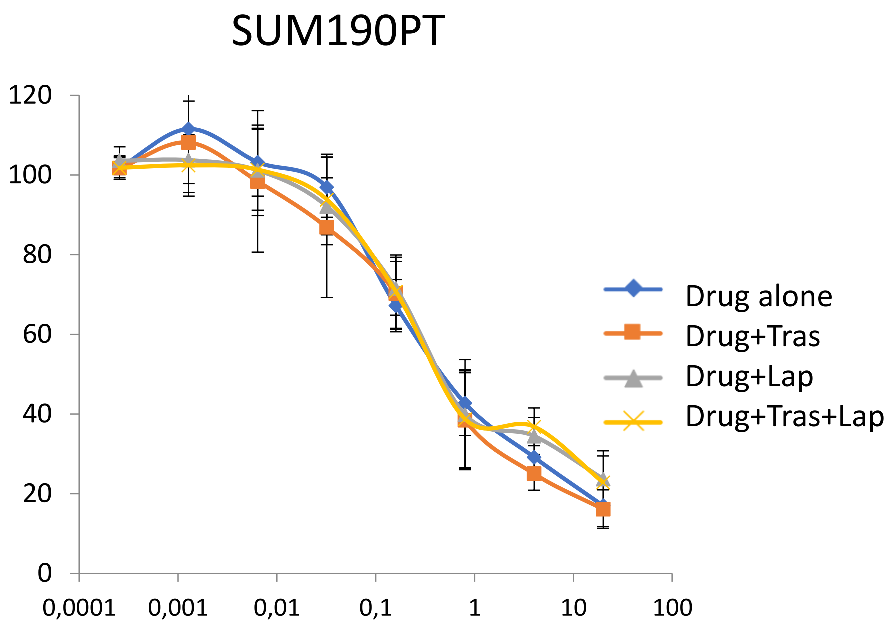

C

Student's t-test *p*-values

	**KPL4**			**SUM190PT**		
Conc (µM)	CUDC vs CUDC+Tras	CUDC vs CUDC+Lap	CUDC vs CUDC+Tras+Lap	CUDC vs CUDC+Tras	CUDC vs CUDC+Lap	CUDC vs CUDC+Tras+Lap
0.000256	0.12	0.33	0.44	0.95	0.30	0.97
0.00128	0.49	0.21	0.09	0.51	0.10	0.05
0.0064	0.70	0.97	0.99	0.50	0.69	0.69
0.032	0.93	0.01	<0.01	0.16	0.21	0.53
0.16	0.58	<0.01	<0.01	0.45	0.21	0.39
0.8	0.45	<0.01	<0.01	0.42	0.62	0.46
4	0.77	0.19	0.10	0.06	0.03	<0.01
20	0.98	0.56	0.52	0.73	0.05	0.07

S3 Fig. In vitro validation of targets from screen. Treatment with increasing doses of the drugs alone and in combination with trastuzumab (10 µg/mL) and lapatinib (0.1 µM) in A) KPL4 cells, and B) SUM190PT cells. C) Table of p-values. Student's t-test p-values calculated between treatment groups at the indicated concentrations. Error bars represent standard deviation of 4 technical replicates repeated in two biological replicates.

S3 Fig.

Danusertib

A

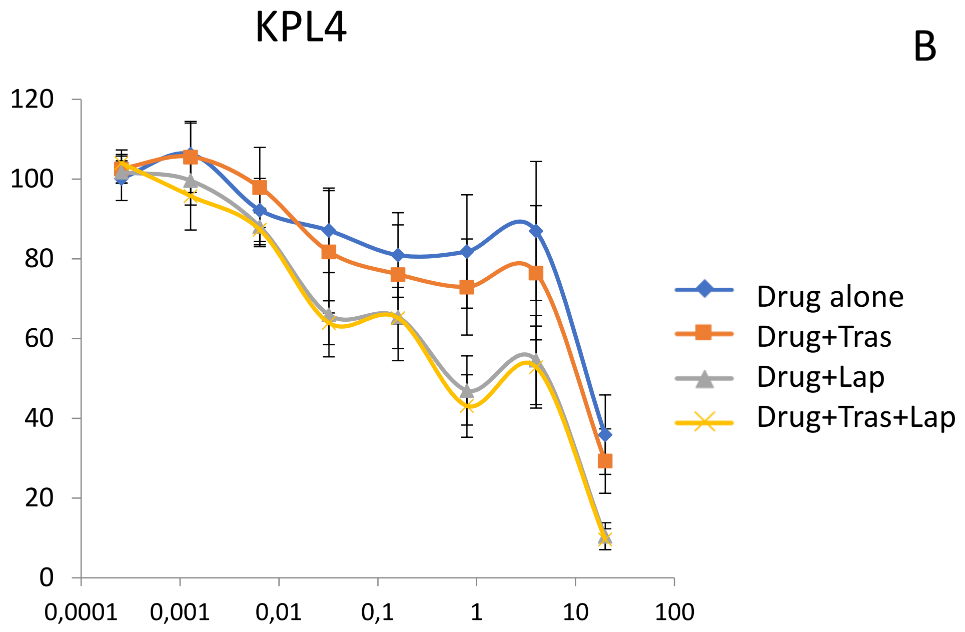

B

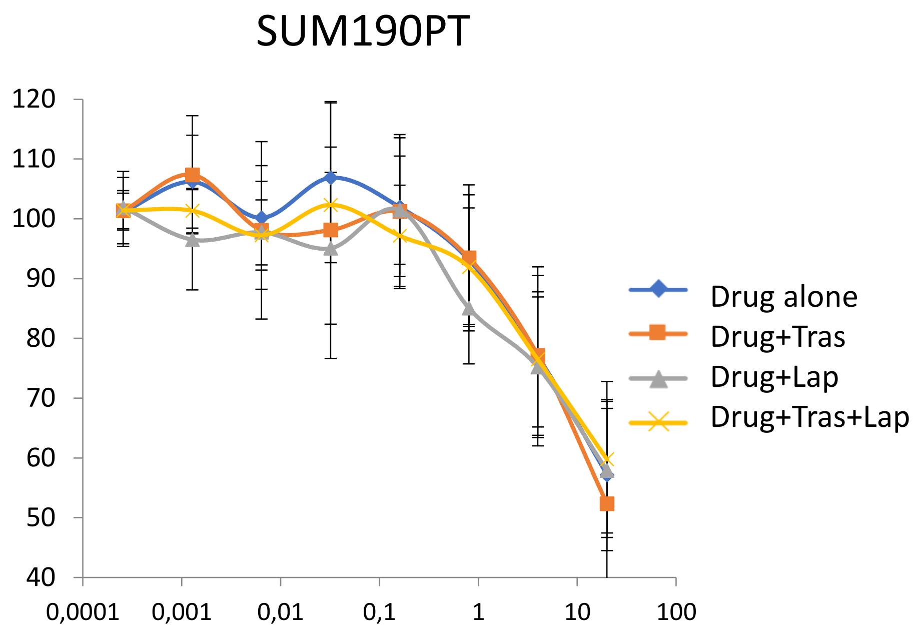

C

Student's t-test *p*-values

	KPL4			SUM190PT		
Conc (µM)	Danu vs Danu+Tras	Danu vs Danu+Lap	Danu vs Danu+Tras+Lap	Danu vs Danu+Tras	Danu vs Danu+Lap	Danu vs Danu+Tras+Lap
0.000256	0.33	0.49	0.12	0.95	0.81	0.92
0.00128	0.87	0.08	0.02	0.80	0.03	0.13
0.0064	0.23	0.22	0.14	0.74	0.51	0.52
0.032	0.43	<0.01	<0.01	0.34	0.08	0.43
0.16	0.42	0.01	<0.01	0.91	0.92	0.36
0.8	0.20	<0.01	<0.01	0.96	0.13	0.82
4	0.24	<0.01	<0.01	0.98	0.79	0.94
20	0.17	<0.01	<0.01	0.53	0.90	0.69

S3 Fig. In vitro validation of targets from screen. Treatment with increasing doses of the drugs alone and in combination with trastuzumab (10 µg/mL) and lapatinib (0.1 µM) in A) KPL4 cells, and B) SUM190PT cells. C) Table of p-values. Student's t-test p-values calculated between treatment groups at the indicated concentrations. Error bars represent standard deviation of 4 technical replicates repeated in two biological replicates.

S3 Fig.

GDC-0941 (Pictilisib)

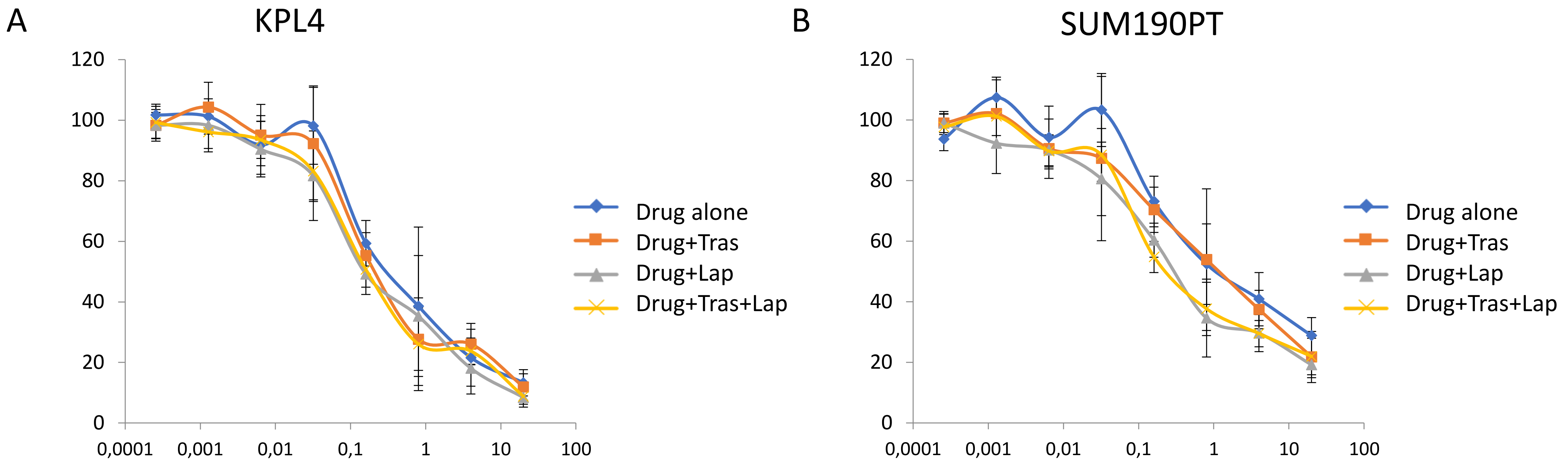

C

Student's t-test *p*-values

	KPL4			SUM190PT		
Conc (µM)	GDC vs GDC+Tras	GDC vs GDC+Lap	GDC vs GDC+Tras+Lap	GDC vs GDC+Tras	GDC vs GDC+Lap	GDC vs GDC+Tras+Lap
0.000256	0.15	0.10	0.29	0.01	0.02	0.07
0.00128	0.40	0.40	0.12	0.26	<0.01	0.08
0.0064	0.52	0.76	0.69	0.47	0.32	0.29
0.032	0.48	0.03	0.02	0.15	<0.01	0.01
0.16	0.30	0.01	0.02	0.50	<0.01	<0.01
0.8	0.29	0.78	0.26	0.88	0.02	0.02
4	0.29	0.45	0.56	0.39	0.01	0.01
20	0.53	0.02	0.03	0.08	<0.01	0.04

S3 Fig. In vitro validation of targets from screen. Treatment with increasing doses of the drugs alone and in combination with trastuzumab (10 µg/mL) and lapatinib (0.1 µM) in A) KPL4 cells, and B) SUM190PT cells. C) Table of p-values. Student's t-test p-values calculated between treatment groups at the indicated concentrations. Error bars represent standard deviation of 4 technical replicates repeated in two biological replicates.

S3 Fig.

OSI-906 (Linsitinib)

A

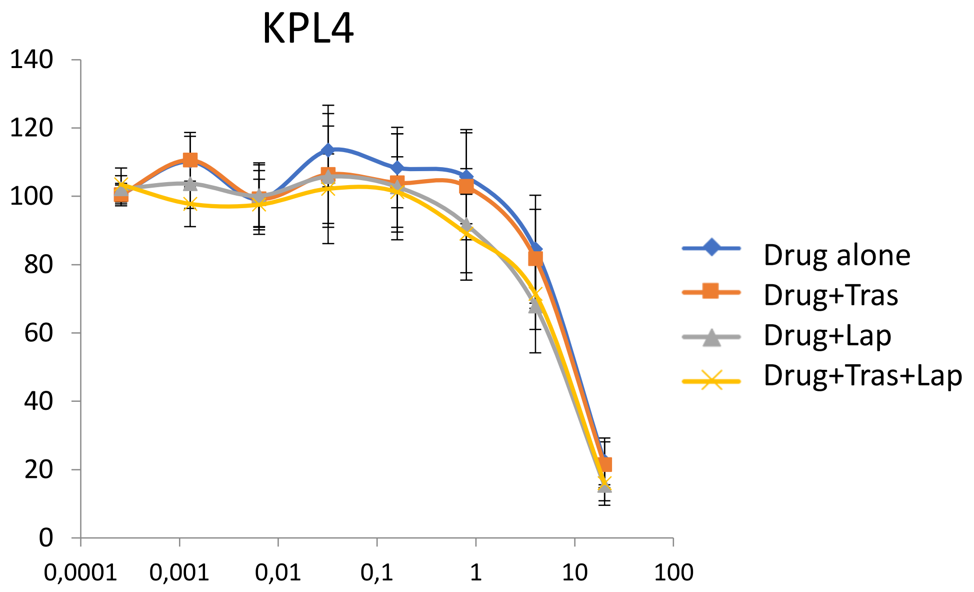

B

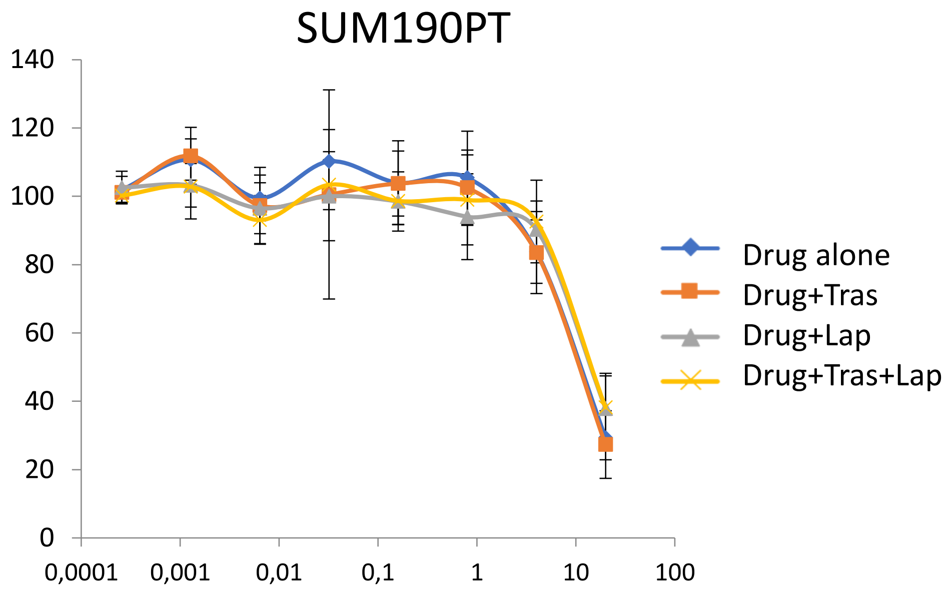

C

Student's t-test *p*-values

	**KPL4**			**SUM190PT**		
Conc (µM)	OSI vs OSI+Tras	OSI vs OSI+Lap	OSI vs OSI+Tras+Lap	OSI vs OSI+Tras	OSI vs OSI+Lap	OSI vs OSI+Tras+Lap
0.000256	0.97	0.36	0.16	0.61	0.77	0.30
0.00128	0.92	0.10	<0.01	0.77	0.03	0.07
0.0064	0.98	0.83	0.68	0.64	0.42	0.09
0.032	0.40	0.25	0.05	0.41	0.09	0.13
0.16	0.51	0.43	0.22	0.97	0.32	0.30
0.8	0.71	0.09	0.02	0.64	0.10	0.35
4	0.72	0.04	0.07	0.96	0.16	0.12
20	0.78	0.04	0.05	0.64	0.06	0.05

S3 Fig. In vitro validation of targets from screen. Treatment with increasing doses of the drugs alone and in combination with trastuzumab (10 µg/mL) and lapatinib (0.1 µM) in A) KPL4 cells, and B) SUM190PT cells. C) Table of p-values. Student's t-test p-values calculated between treatment groups at the indicated concentrations. Error bars represent standard deviation of 4 technical replicates repeated in two biological replicates.

S3 Fig.

Prednisone

A

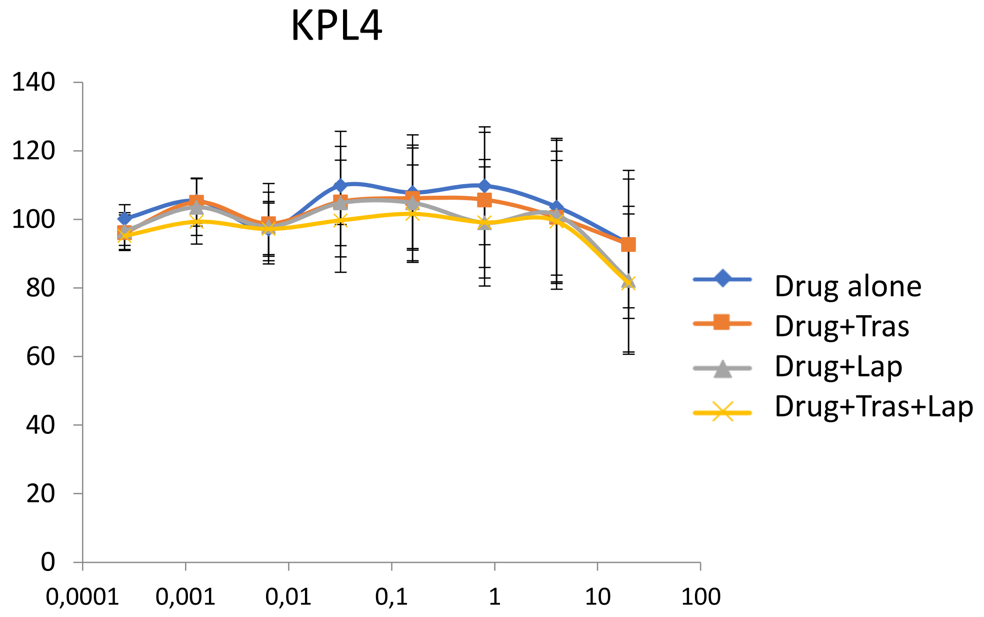

B

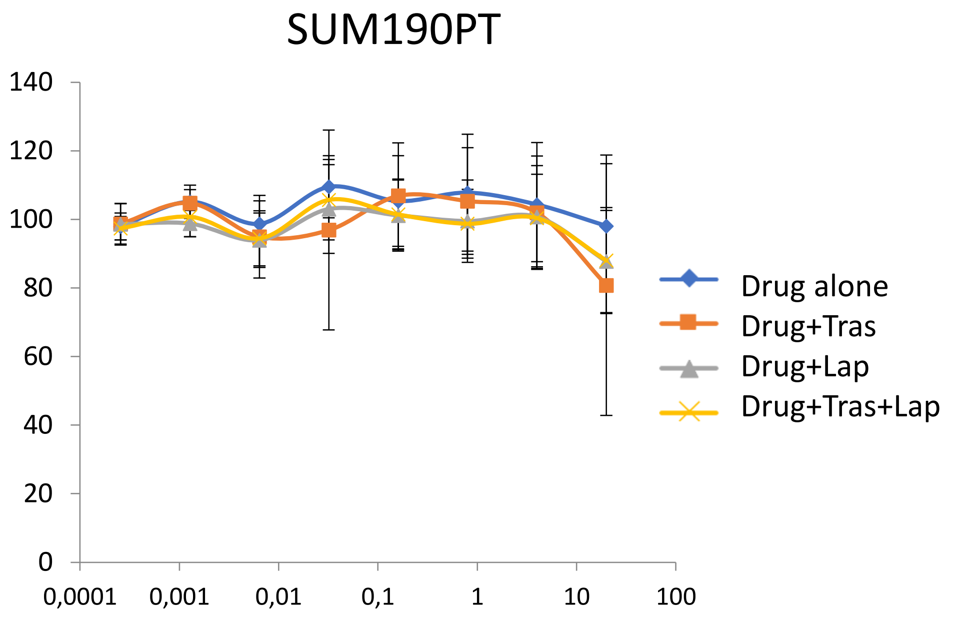

C

Student's t-test *p*-values

	KPL4			SUM190PT		
Conc (µM)	Predni vs Predni+Tras	Predni vs Predni+Lap	Predni vs Predni+Tras+Lap	Predni vs Predni+Tras	Predni vs Predni+Lap	Predni vs Predni+Tras+Lap
0.000256	0.10	0.15	0.01	0.76	0.81	0.78
0.00128	0.90	0.63	0.09	0.88	0.01	0.13
0.0064	0.77	0.88	1.00	0.46	0.22	0.27
0.032	0.58	0.41	0.09	0.26	0.26	0.48
0.16	0.84	0.73	0.44	0.83	0.49	0.52
0.8	0.67	0.25	0.22	0.77	0.28	0.22
4	0.76	0.83	0.66	0.79	0.67	0.63
20	0.98	0.31	0.26	0.27	0.23	0.25

S3 Fig. In vitro validation of targets from screen. Treatment with increasing doses of the drugs alone and in combination with trastuzumab (10 µg/mL) and lapatinib (0.1 µM) in A) KPL4 cells, and B) SUM190PT cells. C) Table of p-values. Student's t-test p-values calculated between treatment groups at the indicated concentrations. Error bars represent standard deviation of 4 technical replicates repeated in two biological replicates.

S3 Fig.

Quisinostat (JNJ-26481585)

A

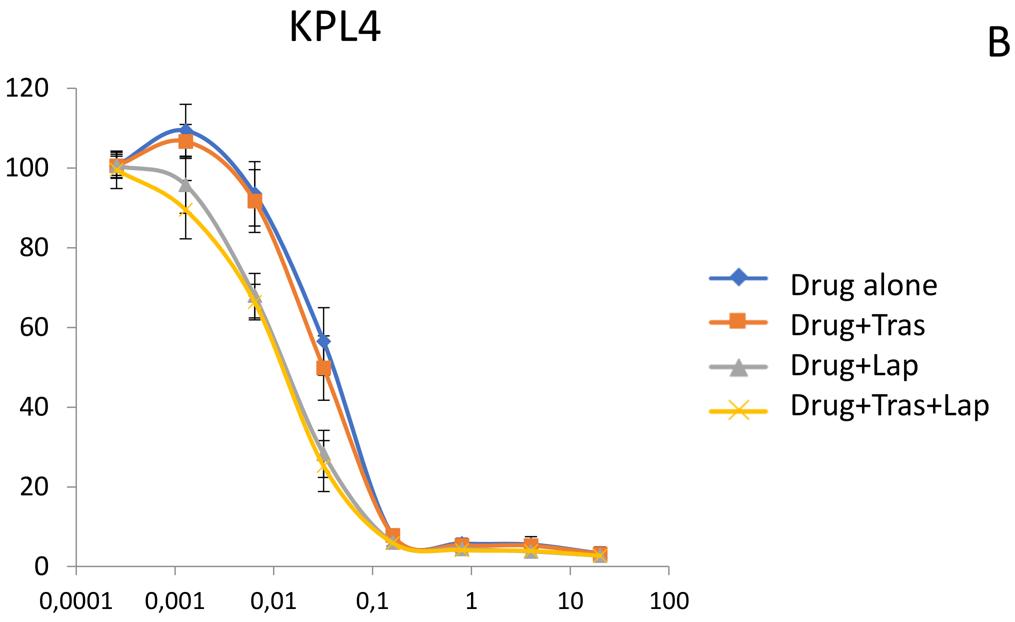

B

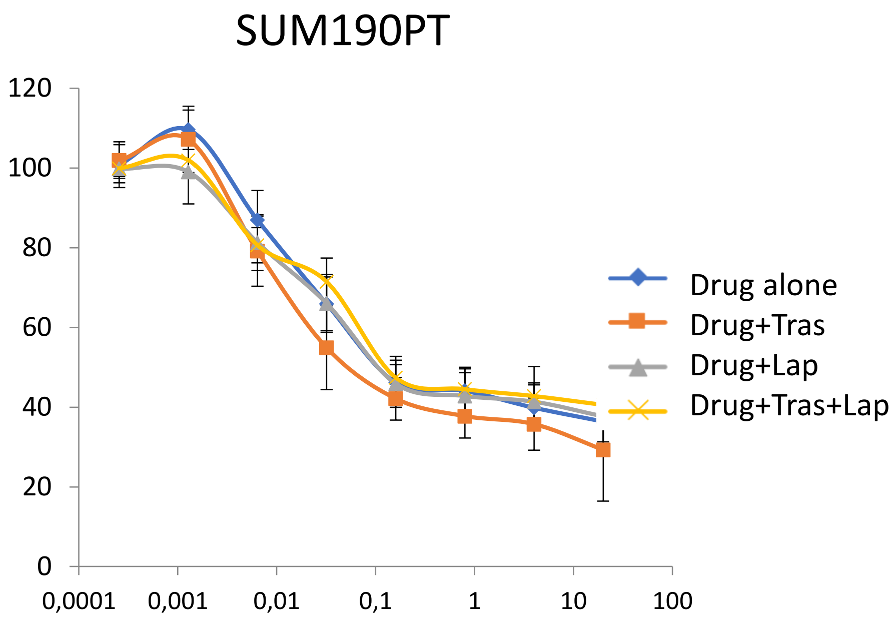

C **Student's t-test *p*-values**

	**KPL4**			**SUM190PT**		
Conc (µM)	Quisino vs Quisino+Tras	Quisino vs Quisino+Lap	Quisino vs Quisino+Tras+Lap	Quisino vs Quisino+Tras	Quisino vs Quisino+Lap	Quisino vs Quisino+Tras+Lap
0.000256	0.98	0.92	0.63	0.67	0.68	0.70
0.00128	0.34	<0.01	<0.01	0.49	0.01	<0.01
0.0064	0.65	<0.01	<0.01	0.08	0.14	0.06
0.032	0.13	<0.01	<0.01	0.03	0.98	0.10
0.16	0.97	<0.01	<0.01	0.13	0.92	0.60
0.8	0.51	0.04	0.01	0.04	0.65	0.88
4	0.77	0.06	0.07	0.20	0.58	0.39
20	0.90	0.52	0.45	0.21	0.75	0.36

S3 Fig. In vitro validation of targets from screen. Treatment with increasing doses of the drugs alone and in combination with trastuzumab (10 µg/mL) and lapatinib (0.1 µM) in A) KPL4 cells, and B) SUM190PT cells. C) Table of p-values. Student's t-test p-values calculated between treatment groups at the indicated concentrations. Error bars represent standard deviation of 4 technical replicates repeated in two biological replicates.
