# Supplementary material for: High-throughput screen in vitro identifies dasatinib as a candidate for combinatorial treatment with HER2-targeting drugs in breast cancer
Source: PLoS One. 2023 Jan 27;18(1):e0280507. doi: 10.1371/journal.pone.0280507 (PMC9882887; doi:10.1371/journal.pone.0280507)
Supplement: S4 Fig — Protein data from tumors treated in vivo with vehicle (Ctr), lapatinib, dasatinib or lapatinib+dasatinib (LapDas) presented in a heatmap sorted on treatment and clustered using pearson average distances on protein expression. (PDF) [file pone.0280507.s004.pdf]

S4 Fig.

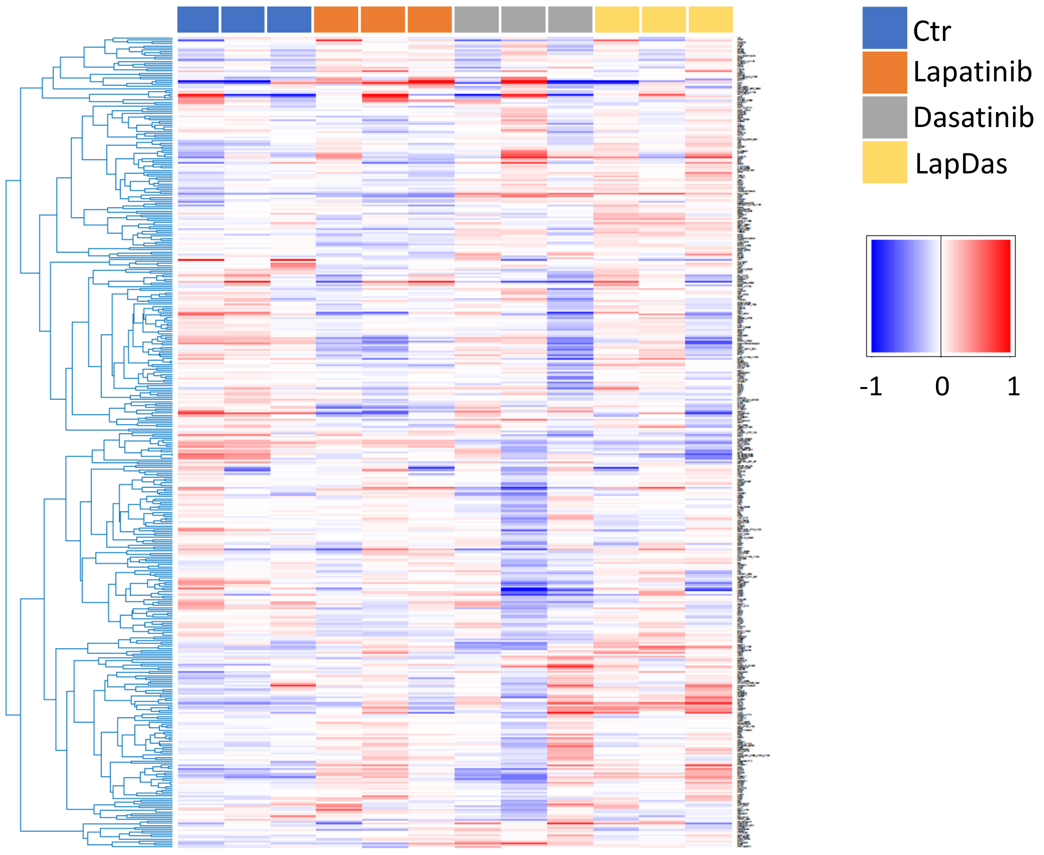

S4 Fig. Heatmap of protein measurements. Protein data from tumors treated in vivo with vehicle (Ctr), lapatinib, dasatinib or lapatinib+dasatinib(LapDas) presented in a heatmap sorted on treatment and clustered using pearson average distance of protein expression.
