# Supplementary material for: High-throughput screen in vitro identifies dasatinib as a candidate for combinatorial treatment with HER2-targeting drugs in breast cancer
Source: PLoS One. 2023 Jan 27;18(1):e0280507. doi: 10.1371/journal.pone.0280507 (PMC9882887; doi:10.1371/journal.pone.0280507)
Supplement: S5 Fig — Protein data from tumors treated with vehicle, lapatinib, dasatinib or lapatinib+dasatinib (LapDas). *p < 0.05, **p < 0.01, Student’s t-test compared to control. Error bars represent standard deviation of protein measurements from three tumors per treatment group. (PDF) [file pone.0280507.s005.pdf]

S5 Fig.

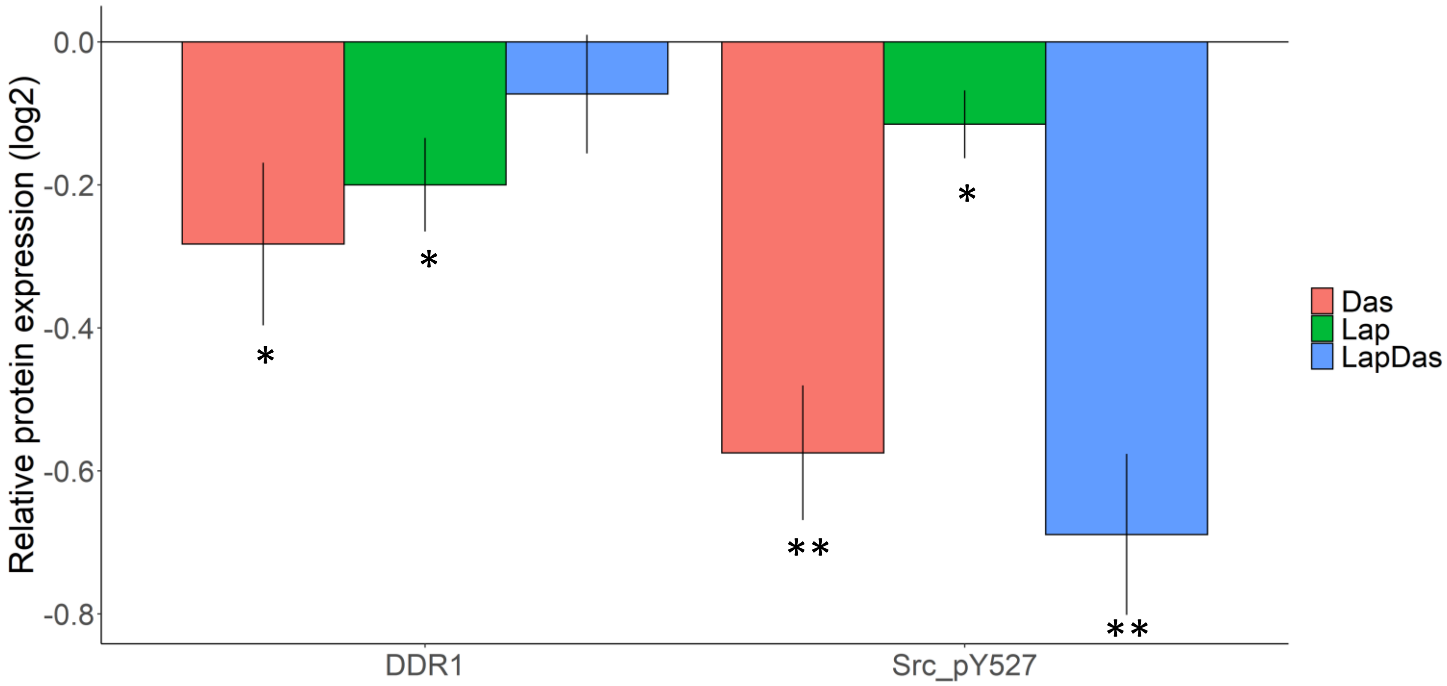

S5 Fig. Protein expression of DDR1 and Src_pY527. Protein data from tumors treated with vehicle, lapatinib, dasatinib or lapatinib+dasatinib (LapDas). *$p < 0.05$, **$p < 0.01$, Student's t-test compared to control. Error bars represent standard deviation of protein measurements from three tumors per treatment group.
