# Supplementary material for: High-throughput screen in vitro identifies dasatinib as a candidate for combinatorial treatment with HER2-targeting drugs in breast cancer
Source: PLoS One. 2023 Jan 27;18(1):e0280507. doi: 10.1371/journal.pone.0280507 (PMC9882887; doi:10.1371/journal.pone.0280507)
Supplement: S1 Table — (PDF) [file pone.0280507.s006.pdf]

S1 Table. List of drugs (n = 278) from the high-throughput screen.

Drug name	Mechanism/Targets
(-)JQ1	Inactive stereoisomer of (+)JQ1
(+)JQ1	BET family inhibitor
15D-PGJ2	Endogenous PPARγ ligand, prostaglandin, NFkB signaling inhibitor
1-methyl-D-tryptophan	Indolamine 2,3-dioxygenase 1 and 2 inhibitor
2-methoxyestradiol	Angiogenesis inhibitor
4-hydroxytamoxifen	Selective estrogen receptor modulator
Abiraterone	P450 17alpha-hydroxylase-17,20-lyase inhibitor
ABT-751	Tubulin inhibitor. Colchicine site binding depolymerizer
Afatinib	EGFR inhibitor
Alisertib	Aurora A kinase inhibitor
Allopurinol	Xanthine oxidase inhibitor
Altretamine	Formaldehyde release, alkylating agent
Alvespimycin	HSP90 inhibitor
Alvocidib	Cdk inhibitor
Amifostine	Cytoprotective adjuvant, free radical scavenging
Aminoglutethimide	Anti-steroid, aromatase inhibitor
Aminolevulinic acid	Photosensitizer
Amonafide	Topoisomerase inhibitor /DNA intercalator
Anagrelide	PDE-3, PLA2 inhibitor
Anastrozole	Aromatase inhibitor
Arsenic(III) oxide	Thioredoxin reductase inhibitor; cytotoxic chemotherapeutic
AT 101	Bcl family inhibitor
AT9283	Aurora A & B, Jak2, Flt, Abl inhibitor
Atorvastatin	HMG CoA reductase inhibitor
Auranofin	Antirheumatic agent
Axitinib	VEGFR, PDGFR, KIT inhibitor
AZ 3146	Mps1 kinase (TTK) inhibitor
Azacitidine	DNMT inhibitor
AZD1152-HQPA	Aurora B inhibitor
AZD1480	JAK1/2, FGFR inhibitor
AZD4547	FGFR inhibitor
AZD7762	Chk1 inhibitor
AZD8055	mTOR inhibitor
Belinostat	HDAC inhibitor
Bendamustine	Nitrogen mustard alkylating agent
Bexarotene	Antineoplastic agent; retinoid specifically selective for retinoid X receptors
BI 2536	PLK1 inhibitor

Bicalutamide	Nonsteriodal antiandrogen
BIIB021	HSP90 inhibitor
Bimatoprost	Prostaglandin analog
Bleomycin	Glycopeptide antibiotic; causes DNA breaks
BMS-754807	IGF1R inhibitor
Bortezomib	Proteasome inhibitor (26S subunit)
Bosutinib	BCR-ABL and src tyrosine kinase inhibitor
Brivanib	VEGFR inhibitor
Bryostatin 1	PKC activator
Buparlisib	PI3K inhibitor, pan-class I
Busulfan	Alkylating antineoplastic agent
Cabozantinib	VEGFR2, Met, FLT3, Tie2, Kit and Ret inhibitor
CAL-101	PI3K inhibitor, p110δ-selective
Camptotechin	Topoisomerase inhibitor
Camptothecin	Topoisomerase I inhibitor
Canertinib	pan-ErbB inhib
Capecitabine	5-FU prodrug. Converted in liver and tumor by 3-step reaction
Carboplatin	Platinum-based antineoplastic agent
Carfilzomib	Proteasome inhibitor (20S subunit)
Carmustine	Alkylating agent
Cediranib	KDR/Flt/VEGFR inhibitor
Celecoxib	Selective COX-2 inhibitor
Chlorambucil	Nitrogen mustard alkylating agent
Chloroquine	Antimalaria agent; chemo/radio sensitizer
CI-994	HDAC inhibitor
Cisplatin	Platinum-based antineoplastic agent
Cladribine	Anti-metabolite; Purine analog
Clofarabine	Anti-metabolite; Purine analog
Clomifene	Selective estrogen receptor modulator
Crizotinib	ALK, c-Met inhibitor
CUDC-101	HDAC & EGFR, Her2 inhibitor
Cyclopamine	Smothened inhibitor
Cyclophosphamide	Alkylating agent
Cytarabine	Anti-metabolite, interferes with DNA synthesis
Dacarbazine	Alkylating agent
Dactinomycin	RNA and DNA synthesis inhibitor
Dactolisib	PI3K/mTOR inhibitor
Danusertib	Aurora, Ret, TrkA, FGFR-1 inhibitor
Daporinad	NAMPT inhibitor
Dasatinib	BCR/Abl, Src,cKit, EphR... Inhibitor
Daunorubicin	Topoisomerase II inhibitor

Decitabine	Nucleoside analog DNA methyl transferase inhibitor
Dexamethasone	Immunosuppresant; glucocorticoid
Dexrazoxone	Chemoprotectant
Docetaxel	Tubulin stabilizer
Doramapimod	p38 inhibitor
Dovitinib	FGFR inhib
Doxorubicin	Topoisomerase II inhibitor
EMD1214063	c-Met inhibitor
Entinostat	HDAC inhibitor
Enzalutamide	AR antagonist
Enzastaurin	PKCbeta inhib
Erismodegib	Smothened (Hh) inhib
Erlotinib	EGFR inhibitor
Estramustine	Alkylating antineoplastic agent
Etoposide	Topoisomerase II inhibitor
Everolimus	mTOR inhibitor
Exemestane	Aromatase inhibitor
Fasudil	Rho kinase, PKA, PKG, PRK inhibitor
Finasteride	type II 5-alpha reductase inhibitor
Fingolimod	S1PR antagonist
Floxuridine	Antimetabolite; Analog of 5-fluorouracil
Fludarabine	Antimetabolite; Purine analog
Fluorouracil	Antimetabolite
Flutamide	Nonsteroidal antiandrogen
Folinic acid	Cytoprotective adjuvant
Foretinib	MET, VEGFR2 inhibitor
Fostamatinib	prodrug of Tamatinib; Syk inhibitor
Fulvestrant	Estrogen receptor antagonist
Galiellalactone	STAT3-DNA interaction inhibitor
Gandotinib	JAK2 inhibitor
GDC-0980	PI3K/mTOR inhibitor
Gefitinib	EGFR inhibitor
Geldanamycin	HSP90 inhibitor
Gemcitabine	Antimetabolite; Nucleoside analog
Goserelin	Gonadotropin releasing hormone superagonist
GSK2126458	PI3K/mTOR inhibitor
Hydroxyurea	Antineoplastic agent
Ibrutinib	Btk inhibitor
Idarubicin	Topoisomerase II inhibitor
Ifosfamide	Nitrogen mustard alkylating agent
Ifosfamide mustard	Alkylating agent

Imatinib	Abl, c-Kit, PDGFRB inhibitor
Imiquimod	Immunomodulatory agent
Indibulin	Tubulin depolymerizer
Iniparib	PARP inhibitor
INK128	mTOR inhibitor
Irinotecan	topoisomerase I inhibitor
Ixabepilone	Microtubule stabilizer, epothilone B analog
KX2-391	non-ATP competitive Src inhibitor
Lapatinib	HER2, EGFR inhibitor
Lasofoxifene	Selective estrogen receptor modulator
Lenalidomide	Immunomodulatory
Lestaurtinib	FLT3, JAK2, TrkA, TrkB, TrkC inhibitor
Letrozole	Aromatase inhibitor
Levamisole	Immunomodulatory agent
Linifanib	VEGFR, PDGFR, CSF-1R, FLT3 inhibitor
Linsitinib	IGF1R, IR inhibitor
Lomustine	Alkylating nitrosourea compound
LY2157299	TGF-B/Smad inhibitor
Masitinib	c-KIT inhibitor
Megestrol	Progestogen
MEK-162	MEK inhibitor
Melphalan	Nitrogen mustard alkylating agent
Mepacrine	Unclear. PLA2 inhibitor. NF-kB inhibitor, p53 activator
Mercaptopurine	Immunosuppresant
Methotrexate	Antimetabolite; Anti-folate agent
Methoxsalen	Psoralen agent
Methylprednisolone	Immunosuppresant
MGCD-265	MET, VEGFR-1, -2, -3, RON, TIE2 inhib
Midostaurin	PKC, KA, S6K and EGFR inhibitor
Mitomycin C	Antineoplastic anatibiotic; DNA crosslinker
Mitotane	Antineoplastic agent
Mitoxantrone	Topoisomerase II inhibitor
MK-0752	gamma-secretase/notch inhibitor
MK1775	Wee1 inhibitor
MK-1775	Wee1 inhibitor
MK-2206	AKT inhibitor
MK-4827	PARP inhibitor
Mocetinostat	HDAC inhibitor (HDAC1 & 2-selective)
Momelotinib	JAK1 & 2 inhibitor
Motesanib	VEGFR, PDGFR, Ret, Kit inhibitor
Navitoclax	Bcl-2 inhibitor

Nelarabine	Nucleoside analog, DNA, RNA synth inhibitor
Neratinib	EGFR inhib, irreversible
Nilotinib	BCR/Abl inhibitor
Nilutamide	Nonsteroidal antiandrogen
Nintedanib	VEGFR, PDGFR, FGFR inhibitor
Nitrogen mustard	Alkylating agent
Nutlin-3	mdm2 inhibitor
NVP-AUY922	HSP90 inhibitor
NVP-BGJ398	FGFR inhibitor
Obatoclax	Bcl2 inhibitor
Olaparib	PARP inhibitor
Omacetaxine	Protein synthesis inhib (80 S ribosome)
OSI-027	mTOR inhibitor
Oxaliplatin	Platinum-based antineoplastic
Paclitaxel	Mitotic inhibitor
Panobinostat	HDAC inhibitor
Patupilone	Tubulin stabilizer
Pazopanib	VEGFR inhib
PD-0332991	Cdk inhibitor (Cdk4/6)
Pemetrexed	Dihydrofolate reductase inhibitor
Pentostatin	Antimetabolite; Purine analog
Perifosine	AKT/PI3K inhibitor
PF 477736	Chk1 inhibitor
PF-00477736	Chk1 inhib
PF-04691502	PI3K/mTOR inhibitor
PF-04708671	p70S6K inhibitor
PF-3845	FAAH inhibitor
PFI-1	Selective chemical probe for BET Bromodomains
Pictilisib	PI3K inhibitor, pan-class I (somewhat p110α-selective)
PIK-75	p110alpha selective PI3K inhibitor
Pilocarpine	Non-selective muscarinic receptor agonist
Pimasertib	MEK inhibitor
Pipobroman	Alkylating agent
Plerixafor	Irreversible CXCR4 antagonist
Plicamycin	RNA synthesis inhibitor
Ponatinib	Broad TK inhibitor
Prednisolone	Immunomodulatory agent
Prednisone	Immunomodulatory agent
Prima-1 Met	p53 activator
Procarbazine	Alkylating agent
Quisinostat	HDAC inhibitor

Quizartinib	FLT3 inhibitor
R935788 aq	Syk inhibitor
Rabusertib	Chk1 inhibitor
RAF265	C-Raf inhib
Raloxifene	Selective estrogen receptor modulator
RD162	AR antagonist
Refametinib	MEK1/2 inhibitor
Regorafenib	B-Raf, c-Kit, VEGFR2 inhibitpr
Ridaforolimus	binds FKBP12, causes inhibition of mTOR
Ruboxistaurin	PKCbeta inhibitor
Rucaparib	PARP inhibitor
Ruxolitinib	JAK1&2 inhibitor
Saracatinib	Src, Abl inhibitor
SB 743921	Eg5/KSP inhibitor
Seliciclib	CDK2/7/9 inhibitor
Selumetinib	MEK inhibitor
Serdemetan	HDM2-p53 antagonist
Sirolimus	binds FKBP12, causes inhibition of mTOR
SNS-032	Cdk inhibitor
Sonolisib	PI3K inhibitor, pan-class I. Irreversible
Sorafenib	B-Raf, FGFR-1, VEGFR-2 & -3, PDGFR-?, c-KIT, and Flt3 inhib
Sotrastaurin	PKC inhibitor
StemRegenin 1	AHR antagonist, stem cell regenerating
Streptozocin	Glucosamine-nitrosourea agent
S-trityl-L-cysteine	Eg5 inhibitor
Sunitinib	Broad TK inhibitor
Tacedinaline	HDAC inhibitor
Tacrolimus	Binds FKBP12, causes inhibition of calcineurin
TAK-733	MEK inhibitor
TAK-901	Aurora B inhibitor
Tamatinib	Syk inhibitor, active substance of Fostamatinib
Tamoxifen	Estrogen receptor antagonist
Tandutinib	FLT3, PDGFR, KIT inhibitor
Tanespimycin	HSP90 inhibitor
Tarenflurbil	Gamma-secretase inhibitor
Temozolomide	Alkylating agent
Temsirolimus	binds FKBP12, causes inhibition of mTOR
Teniposide	Topoisomerase II inhibitor
TGX-221	p110beta selective PI3K inhibitor
Thalidomide	Immunosuppresant
Thioguanine	Antimetabolite; Purine analog

Thiotepa	Alkylating agent
Tipifarnib	Farnesyltransferase inhibitor
Tivantinib	c-Met inhibitor
Tivozanib	VEGFR1, 2, 3, c-Kit, PDGFRB inhibitor
Tofacitinib	JAK3, JAK2(V617F) inhib
Topotecan	Topoisomerase I inhibitor
Toremifene	selective estrogen receptor modulator
Tosedostat	Aminopeptidase inhibitor
Trametinib	MEK1/2 inhibitor
Tretinoin	Retinoic acid receptor agonist
Triethylenemelamine	Antineoplastic agent
Tubacin	HDAC6 inhibitor
Tubastatin A	HDAC6 inhibitor
UCN-01	PKCbeta, PDK1, Chk, Cdk2 inhibitor
Uracil mustard	Alkylating agent
Valproic acid	HDAC inhibitor
Valrubicin	Topoisomerase II inhibitor
Vandetanib	VEGFR,EGFR, RET inhibitor
Varespladib	Secretory phospholipase A2 inhibitor
Vatalanib	VEGFR-1 & -2 inhibitor
Veliparib	PARP inhibitor
Vemurafenib	B-Raf(V600E) inhibitor
VER 155008	HSP70 inhibitor
Vinblastine	Mitotic inhibitor
Vincristine	Mitotic inhibitor
Vinorelbine	Antimitotic chemotherapy
Vismodegib	Smothened (Hh) inhibitor
Volasertib	PLK1 inhibitor
Vorinostat	HDAC inhibitor
VX-11E	ERK1 & 2 inhibitor
XAV-939	Tankyrase-1 and -2
XL147	PI3K inhibitor. Pan-class I
XL-147	PI3K inhibitor
XL765	mTOR/PI3K inhibitor
YM155	Survivin inhibitor
Zoledronic acid	Bisphosphonate
