# Supplementary material for: High-throughput screen in vitro identifies dasatinib as a candidate for combinatorial treatment with HER2-targeting drugs in breast cancer
Source: PLoS One. 2023 Jan 27;18(1):e0280507. doi: 10.1371/journal.pone.0280507 (PMC9882887; doi:10.1371/journal.pone.0280507)
Supplement: S2 Table — (PDF) [file pone.0280507.s007.pdf]

S2 Table. Top sensitizing drugs from the high-throughput screen and their molecular targets.

Drug	Type of drug
Carboplatin	Chemotherapy. Interferes with DNA repair
CUDC-101	Inhibitor of HDAC, EGFR, HER2
Danusertib	Inhibitor of Aurora A, B, C kinases, Bcr-Abl, c-RET, TrkA, FGFR-1
Dasatinib	Inhibitor of Src family, Bcr-Abl, cKit, EphR
Linsitinib (OSI-906)	Inhibitor of IGF-1R and IR
Navitoclax	Inhibitor of Bcl-x_L, Bcl-2
Pictilisib (GDC-0941)	Inhibitor of PI3K
Prednisone	Immunomodulatory drug
Quisinostat (JNJ-26481585)	Inhibitor of HDAC

The drugs are presented in alphabetical order.
