# Supplementary material for: High-throughput screen in vitro identifies dasatinib as a candidate for combinatorial treatment with HER2-targeting drugs in breast cancer
Source: PLoS One. 2023 Jan 27;18(1):e0280507. doi: 10.1371/journal.pone.0280507 (PMC9882887; doi:10.1371/journal.pone.0280507)
Supplement: S3 Table — (PDF) [file pone.0280507.s008.pdf]

S3 Table. IPA signaling pathways.

	Z-score		
**Ingenuity Canonical Pathways**	**Das vs Ctr**	**Lap vs Ctr**	**Lapdas vs Ctr**
HER-2 Signaling in Breast Cancer	-1.46	-1.46	-0.66
ERK/MAPK Signaling	-2.67	-1.67	-2.33
Negative z-score (decreased activation)			

Selected signaling pathways that are predicted in IPA to be affected due to the protein level regulations upon treatment. A negative Z-score indicates inhibition. Das = dasatinib; Ctr = control; Lap = lapatinib; LapDas = lapatinib + dasatinib.
