# Supplementary material for: High-throughput screen in vitro identifies dasatinib as a candidate for combinatorial treatment with HER2-targeting drugs in breast cancer
Source: PLoS One. 2023 Jan 27;18(1):e0280507. doi: 10.1371/journal.pone.0280507 (PMC9882887; doi:10.1371/journal.pone.0280507)
Supplement: S4 Table — (PDF) [file pone.0280507.s009.pdf]

S4 Table. IPA diseases and functions.

	Z-score			
**Diseases and Functions Annotation**	**Das vs Ctr**	**Lap vs Ctr**	**Lapdas vs Ctr**	**Lapdas vs Lap**
Apoptosis	1.65	1.96	0.53	-3.21
Necrosis	1.41	1.55	0.99	-2.84
Cell survival	-3.10	-2.83	-1.12	2.46
Cell viability of tumor cell lines	-2.49	-2.52	-1.05	1.60
Positive z-score (increased activation)				
Negative z-score (decreased activation)				

Selected diseases and functions that are predicted in IPA to be affected due to the protein level regulations upon treatment. A positive Z-score indicates activation, while a negative Z-score indicates an inhibition. Das = dasatinib; Ctr = control; Lap = lapatinib; LapDas = lapatinib + dasatinib.
